# Potential Drug Development Candidates for Human Soil-Transmitted Helminthiases

**DOI:** 10.1371/journal.pntd.0001138

**Published:** 2011-06-07

**Authors:** Piero Olliaro, Jürg Seiler, Annette Kuesel, John Horton, Jeffrey N. Clark, Robert Don, Jennifer Keiser

**Affiliations:** 1 UNICEF/UNDP/World Bank/WHO Special Programme on Research and Training in Tropical Diseases (TDR), World Health Organization, Geneva, Switzerland; 2 ToxiConSeil, Riedtwil, Switzerland; 3 Tropical Projects, Hitchin, United Kingdom; 4 JNC Consulting Services LLC, Pittsboro, North Carolina, United States of America; 5 Drugs for Neglected Diseases initiative (DNDi), Geneva, Switzerland; 6 Department of Medical Parasitology and Infection Biology, Swiss Tropical and Public Health Institute, Basel, Switzerland; 7 University of Basel, Basel, Switzerland; McGill University, Canada

## Abstract

**Background:**

Few drugs are available for soil-transmitted helminthiasis (STH); the benzimidazoles albendazole and mebendazole are the only drugs being used for preventive chemotherapy as they can be given in one single dose with no weight adjustment. While generally safe and effective in reducing intensity of infection, they are contra-indicated in first-trimester pregnancy and have suboptimal efficacy against *Trichuris trichiura*. In addition, drug resistance is a threat. It is therefore important to find alternatives.

**Methodology:**

We searched the literature and the animal health marketed products and pipeline for potential drug development candidates. Recently registered veterinary products offer advantages in that they have undergone extensive and rigorous animal testing, thus reducing the risk, cost and time to approval for human trials. For selected compounds, we retrieved and summarised publicly available information (through US Freedom of Information (FoI) statements, European Public Assessment Reports (EPAR) and published literature). Concomitantly, we developed a target product profile (TPP) against which the products were compared.

**Principal Findings:**

The paper summarizes the general findings including various classes of compounds, and more specific information on two veterinary anthelmintics (monepantel, emodepside) and nitazoxanide, an antiprotozoal drug, compiled from the EMA EPAR and FDA registration files.

**Conclusions/Significance:**

Few of the compounds already approved for use in human or animal medicine qualify for development track decision. Fast-tracking to approval for human studies may be possible for veterinary compounds like emodepside and monepantel, but additional information remains to be acquired before an informed decision can be made.

## Author Summary

There are few drugs - none ideal - for the treatment and control of gastrointestinal helminths (soil-transmitted nematodes) which, as chronic infections jeopardize children's growth, learning and ultimately individual, community and country development. Drugs for helminths are not attractive in human medicine, but are lucrative in animal health. Traditionally, investment in veterinary medicines has benefited humans for these diseases. With modern regulations an approved veterinary medicine can be tested in humans with little adaptation, reducing time and cost of development. We searched for products that could easily be transitioned into humans, having the necessary characteristics for use in communities exposed to these infections. A limited number of candidates met the main criteria for selection. We provide here a detailed analysis of two veterinary products, emodepside and monepantel, and nitazoxanide, which is approved for human use. In addition we include a less detailed analysis of all products examined, and the criteria on which the analysis was based. It is clear that the pipeline of easily obtainable human anthelminthics remains extremely limited, and further efforts are needed to find replacements for the inadequate number of products available today.

## Introduction

Soil-transmitted helminthiasis (STH) is caused primarily by four species of nematode worms, *Ancylostoma duodenale* and *Necator americanus* (hookworms), *Ascaris lumbricoides* (roundworm), and *Trichuris trichiura* (whipworm), that parasitize the human gastrointestinal tract [1]. Some 2–3 billion people are thought to have active infections and billions more are at risk of infection [2]–[4]. STH is estimated to be responsible for the loss of 39 million disability adjusted life years (DALYs) annually [2] but the burden of disease is currently being re-evaluated [5].

There are only four drugs recommended by the World Health Organization (WHO) for STH, and they have been in use for several decades: the two benzimidazole carbamates (BZs) albendazole and mebendazole, levamisole, and pyrantel pamoate [6], [7]. While STH related morbidity can be controlled through chemotherapy, various problems need to be faced as anthelmintics are increasingly deployed in mass drug administration (MDA) programs [6]. For practical reasons MDA requires a single drug administration to all subjects without prior diagnosis or checking for contra-indications.

For this reason, the BZs are preferred over levamisole and pyrantel (which require weight-based dosing and are also intrinsically less potent). However, the BZs are not perfect drugs either: first, the efficacy against some of the STHs (especially *T. trichiura*) is suboptimal when delivered as a single dose [8]; second the BZs are contra-indicated in early pregnancy, which may go unnoticed or unreported in the first trimester; and finally, the wide-spread coverage with a single class of compounds exposes parasites to selective pressure potentially leading to resistance, which has already occurred widely in veterinary practice [9], [10]. Therefore, there is a pressing need for concerted efforts to discover and develop the next generation of anthelmintic drugs and for drug combinations. The main drivers are risk of emerging BZ resistance, the limited spectrum of activity and the contraindications of the current drugs.

Discovering and developing new drugs (Research & Development, R&D) is a complicated, expensive, risky and time-consuming endeavour. For a new drug to be granted marketing authorization in humans, it must be developed following strict regulatory requirements [11]. Clinical development is also the most expensive part of R&D [11]. Therefore, the decision to move a compound from the discovery into the development stage must be carefully considered and based on sound science and cost considerations.

Our goal is to identify and evaluate potential development candidates for STHs to assess whether all data required to inform the decision to initiate development are available or what additional data are needed. Compounds registered for veterinary medical use by the US Food and Drug Administration (FDA), European Medicines Agency (EMA) and other regulatory bodies have extensive safety, pharmacokinetics (in some cases) and efficacy data derived in animals. Therefore, these should have the potential for accelerated transition into human use.

Almost all products currently available for human helminth diseases have been transitioned from veterinary/animal health companies since the 1950's [7], [12]. The average transition time was 3 years, but for older veterinary drugs, this was longer because of the need to conduct additional studies (e.g. safety, pharmacology) to satisfy modern requirements.

Modern rules for veterinary medicine licensure means that data available now are essentially equivalent to those required for human medicines; therefore transitioning can be achieved earlier. However, criteria for veterinary medicine anthelmintic efficacy are much more stringent than current human requirements, e.g. generally require an efficacy defined as 90% or greater clearance of the target organism [12], [13].

The underlying approach for this analysis was first to assess candidates based on publicly available information (through US Freedom of Information (FoI) statements, European Public Assessment Reports (EPAR), scientific meetings, publications, patents, etc) that could be summarized and shared. For the compounds that emerge as promising from this initial assessment, further information will be sought by directly contacting the relevant data owner for additional confidential data as appropriate. This will allow identification and analysis of the missing elements to permit an informed decision to be made on taking the compound forward and for planning the additional experiments that are required for human registration.

This paper summarizes the general findings and more specific information on two veterinary anthelmintics (monepantel, emodepside) and on nitazoxanide, a licensed antiprotozoal drug with evidence for anthelminthic activity, compiled from an analysis of publicly available information in the EMA EPAR and FDA registration files. Complete reviews with these data are provided as supplementary data (see below). None of these has been assessed for STH from a human drug development perspective. These summaries are made available to help define and stimulate decisions by potential researchers and drug developers, identify additional investigations that may be needed for an informed decision, as well as creating development partnerships.

## Materials and Methods

### Search strategy

Two searches were performed in parallel:

- We searched PubMed (http://www.ncbi.nlm.nih.gov) up to March 1, 2010 and contacted experts to identify potential drug development candidates for treating infections with soil transmitted helminths. For the electronic search the terms “chemotherapy”, “drug” or “anthelmintic”, in combination with “in vivo” and “ascariasis”, “*Ascaris lumbricoides*”, “hookworm”, “*Ancylostoma duodenale*”, “*Necator americanus*”, “trichuriasis”, “*Trichuris trichiura*”, “soil-transmitted helminths”, “soil-transmitted helminthiases”, or “nematode” were used. The search was restricted to publications over the past 20 years.
- Marketed veterinary drugs (U.S. and/or ex-U.S.) were also examined and categorized by the various chemical classes. Documents reviewed for this exercise included the U.S. FoI statements prepared by the veterinary drug companies and approved by the FDA, EMEA and other ex-U.S. regulatory body registration files, company product information, patents, scientific meeting abstracts and published literature and knowledge gathered by one of the authors (JNC) over 30 years in the animal health drug industry.

### Assessment criteria

The candidate compounds for the searches were assessed against a product profile that had been previously generated following discussions at several meetings of experts at WHO and elsewhere (see proposed target product profile (TPP) which is provided in detail in Appendix S1). The assessment criteria used for the analysis contain the key points of the TPP and required the drug to be potentially:

1. Safe for mass drug administration (MDA)
  as well tolerated as in-use BZs albendazole and mebendazole
  ideally capable of being given to pregnant women (ensuring maximum use in affected populations)
2. Affordable in the context of MDA in endemic countries
3. Achieving desired effect in a single dose (maximum two doses in one day)
4. Simple to dose (not requiring complex measurements to deliver the drug)
5. And additionally:
  Spectrum to cover *A. lumbricoides*, both *N. americanus* and *A. duodenale*, *T. trichiura* (and also possibly *S. stercoralis*)
  Minimally or not absorbed (if significantly absorbed, safety margins must be high)
  No cross-resistance to existing drugs

To identify compounds amenable to rapid development, we aimed for compounds that are already in human or veterinary use or that had gone through extensive animal testing in their development as veterinary drugs.

## Results

### Searches and assessment of potential drug candidates identified

The electronic search on PubMed yielded 299 hits. After discarding duplicate publications and studies outside our scope (e.g. molecular papers) and veterinary drugs (as these were already identified in our parallel search as described below), 25 potential drug candidates remained. The majority of these were natural product compounds and with the exception of tribendimidine, none of these had undergone extensive animal testing, and hence were not considered further. Through expert consultation an additional compound was identified (nitazoxanide).

In addition, we identified several primary anthelmintics used in veterinary medicine today. These include various representatives from the macrocyclic lactones (MLs, avermectins and milbemycins, including some experimental compounds that did not reach the market), BZs, depsipeptides, paraherquamides, hexahydropyrazines, tetrahydropyrimidines, imidathiazoles, amino-acetonitriles, salicylanilides, phenylsulfonamides, biphenylsulfides and miscellaneous compounds. Although there are some compounds (e.g. phenothiazines) that have been used in veterinary medicine, these are very old drugs and were not thought to be worth including here. A representative of each class is summarized in Table 1; details are provided in Appendix S2. Additional details on approved animal health compounds identified, including compound class, generic name, chemical structure, current supplier, patent approval, U.S. approval, mode of action, more specifics on parasite claims and efficacy/resistance, dose rates, more on safety and toxicity issues and an overall assessment of current use in veterinary medicine.

**Table 1:** Summary of approved veterinary anthelmintics.

Compound class	Drug (example)	Current supplier	Efficacy	Safety and toxicity
Avermectin	Ivermectin	Merial, Ltd	Broad spectrum nematocide including immature filarial worms	Wide safety margin at use level; no reproductive safety issues
Milbemycin	Moxidectin	Pfizer	Broad spectrum nematocide including immature filarial worms	Wide safety margin at use level; no reproductive safety issues
Benzimidazoles	Albendazole	GSK	Broad spectrum anthelmintic; widespread resistance amongst veterinary parasites	Generally safe but teratology issues known in the compound class in first trimester
Depsipeptide	Emodepside	Bayer	Approved for ascarids and hookworms in cats	Wide safety margin with approved drug
Paraherquamide	Derquantel	Pfizer	Most GI nematodes in sheep and active against those resistant to other nematocides	Derquantel is an approved veterinary drug, but paraherquamide A is quite toxic in dogs and mice
Hexahydroxy-pyrazines	DEC	Generic	Anti-filarial: immature stages	Contraindicated for dogs infected with heartworm
	Piperazine	Dow	Ascarids (birds and swine) and *Oesophagostomum* spp. in swine	Safe drug; oral rat LD_50_ 1900 mg/kg
	Praziquantel	Bayer	Tapeworms, broad spectrum trematocidal activity except *Fasciola* spp.	Wide safety margin at use level; safe for use in pregnant dogs
Tetrahydro-pyrimidines	Pyrantel pamoate	Pfizer	Ascarids and hookworms in dogs; GI nematodes in horses	Wide safety margin at use level; no reproductive safety issues
Imidazo-thiazoles	Levamisole	Janssen	Broad spectrum nematocide, excluding filarial worms; widespread resistance amongst sheep abomasal parasites	Wide safety margin at use level; no reproductive safety issues
Amino-aceto-nitriles	Monepantel	Novartis	Broad spectrum activity against sheep GI nematodes	No contraindications and no reproductive effects observed in rats or rabbits
Salicylanilides	Closantel	Janssen	Sheep liver fluke and *Haemonchus contortus*	Only for use in male sheep, overdose might cause blindness
	Rafoxanide	Merial, Ltd	Cattle, sheep liver fluke and GI nematodes	Some teratology noted in safety studies in rats and rabbits
Phenyl-sulfonamide	Clorsulon	Merial, Ltd	Cattle adult and immature liver fluke	Wide safety margin
Biphenylsulfides	Bithionol	Monsanto	Rumen and liver flukes, tapeworm	Sulfoxide form toxic at 280 mg/kg.Contraindicated for animals with renal disease
	Febantel	Bayer	Canine intestinal tract nematodes including *Trichuris vulpis*	Potentially teratogen in first trimester as pro-benzimidazole
Miscellaneous	Nitroscanate	Novartis	Canine intestinal nematodes	Safe to give to pregnant animals; can cause vomiting in some dogs on dosing
	Nitroxynil	Generic	Cattle and sheep flukes and limited GI nematode spectrum	No safety issues reported

We did not further consider the avermectin class (which comprise a large number of animal health registered compounds, such as doramectin, eprinomectin, ivermectin and selamectin and agrochemical-registered compounds such as abamectin and emamectin) to be potentially interesting drug development candidates, as these drugs would likely be cross resistant to ivermectin-resistant parasites. In addition, ivermectin is characterized by low efficacy against hookworms [14], [15]. The aforementioned compounds are all very similar structurally and act by the same mode of action, and therefore are unlikely to offer a clear advantage over ivermectin in terms of efficacy or resistance. No further search was undertaken for moxidectin, a milbemycin macrocyclic lactone, as this drug is under development for systemic helminths (onchocerciasis) in humans. The physico-chemical characteristics of this molecule result in pharmacokinetic advantages over ivermectin (longer residence time, larger volume of distribution). Though milbemycin oxime, another compound in this class, was effective in the treatment of ascarids and hookworms in naturally infected cats [16] and in dogs (Interceptor® product label) and is approved for *Trichuris vulpis* in dogs (Interceptor® product label), only moderate egg reduction rates were observed in baboons infected with *T. trichiura* [17]. Further, this compound is only registered for use in companion animals so much of the data generated for a food producing animal that would accelerate any human health program would not be readily available.

The BZs represent a wide variety of molecules developed by several animal health companies and launched mostly in the 1960's and early 1970's for livestock, horses and companion animals. As mentioned above, albendazole and mebendazole are the most widely used drugs against STH today. The BZs act as inhibitors of tubulin formation, affecting cell synthesis and function [18], [19]. The main disadvantage is that, as a class, the BZs are teratogens in animals and are contraindicated for use in the first trimester of pregnancy. Additionally, there is concern that their widespread use in public health programmes in highly endemic countries will result in helminth resistance, just as seen in the veterinary field a few years after their introduction. Before selecting any of the other BZs identified (fenbendazole, flubendazole, oxfendazole, oxibendazole, thiabendazole and netobimin) for development for STHs, any advantage over albendazole or mebendazole in terms of potential cross-resistance, improved efficacy profile or contraindications, would have to carefully considered. For example, fenbendazole given at doses of 30–50 mg/kg only achieved a cure rate of 28.6% in 28 Korean patients with *T. trichiura* respectively [20]. Finally, oxfendazole and flubendazole are currently being investigated for treatment of systemic helminth infections in humans by the NIH and BMGF, and no publicly available information in humans exists for oxibendazole, which had been in clinical development for STH by SmithKlineBeecham/GlaxoSmithKline until about 2003.

Paraherquamide A is a natural product produced by *Penicillium paraherquei* which was discovered in 1981 [21]. It was evaluated by Merck in the late 1980's and a small chemistry effort was conducted to produce analogs [22]. Paraherquamide A was found to have outstanding broad spectrum nematocidal activity against various sheep gastro-intestinal nematodes [23]. It is a nicotinic antagonist that blocks depolarization in muscles and induces a rapid paralysis of the mid-body of the parasite [24]. However, it was severely toxic in mice and dogs, which prevented its development [25], as these species are the standard models for safety studies. In addition, poor activity was observed against *T. vulpis* in dogs [25]. UpJohn, later Pfizer, conducted semi-synthetic medicinal chemistry on Paraherquamide A [26] and eventually identified derquantel as a safer but still effective compound against sheep gastrointestinal parasites. Derquantel was noted, however, to cause lethality in horses [27] and was not pursued for this species. This product is being developed as a sheep product in Australia and New Zealand in combination with abamectin [28]. It remains to be confirmed whether derquantel offers improved efficacy against *Trichuris* spp. In addition, a thorough evaluation of potential toxicity of derquantel or any metabolites will have to be done prior to any administration to humans, acknowledging the history of this compound class. This issue lowered the priority for this compound in our evaluation.

No new compounds were identified within the hexahydropyrazine and imidazothiazole classes. Many of the hexahydropyrazines (DEC, piperazine, praziquantel and epsiprantel) and the imidazothiazole levamisole have been used for many years in human health. Similarly, the tetrahydropyrimidine class of neuromuscular blocking agents, such as pyrantel, has been used for decades in human health [8], [29], and the related molecule morantel would not offer any advantage over pyrantel.

Amidantel (BAY d 8815), a precursor of tribendimidine, was evaluated by Bayer in late 1970's. It showed efficacy against hookworms and ascarids in dogs with a single oral dose of 25 mg/kg [30], including *Toxacara canis*, which was completely eliminated by a single 10 mg/kg oral treatment. Early studies showed the compound acted as an acetylcholine agonist [31]. The compound was not marketed as a veterinary product as the drug had to be given twice on two consecutive days, which was a great disadvantage in the face of other existing anthelmintics for companion animals [32]. As tribendimidine, a symmetrical diamidine derivative of amidantel, is marketed in China for STH [33] and being pursued for human use, amidantel was not considered as a candidate from our analysis.

The salicylanilides (closantel, niclosamide, oxyclozanide, rafoxanide), the phenylsulfonamide clorsulon, the biphenylsulfides bithionol and febantel, and nitroscanate and nitroxynil are structurally similar with all containing one or more phenyl groups with halide or phenolic hydroxyls and/or nitro group substitutions. They are among the older anthelmintics developed for veterinary medicine and are still used, although safer and broader spectrum parasiticides take precedence except where price is more of a priority. They generally act as uncouplers of oxidative phosphorylation and so would not be prime candidates for human development without a thorough toxicology evaluation. In addition, several of these drugs (e.g. bithionol, clorsulon, rafoxanide) are only active against the trematode *Fasciola hepatica* [34], [35] and do not possess nematocidal activity.

**Figure 1:**
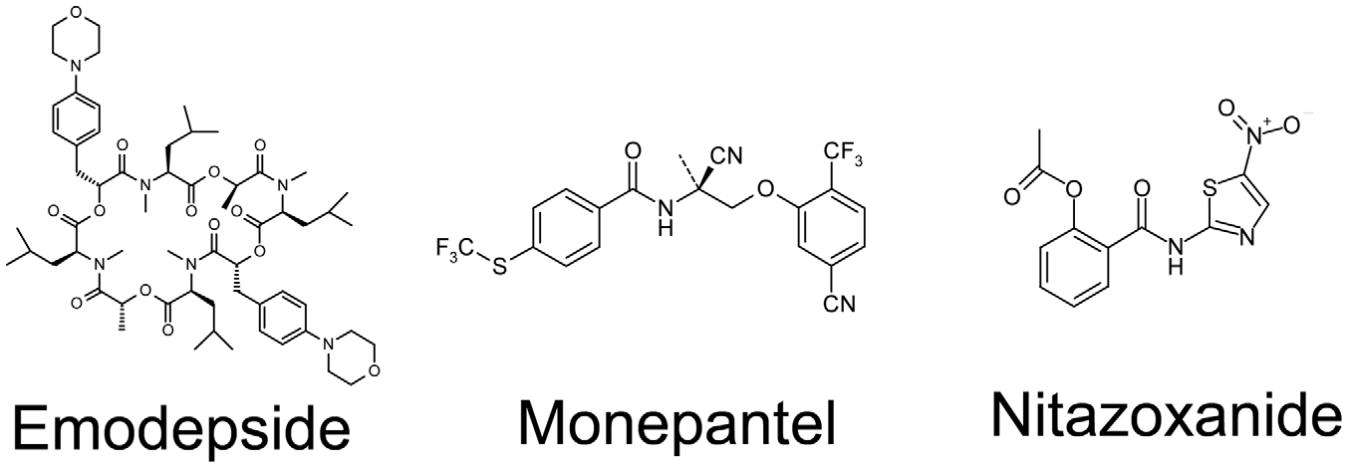
Chemical structures of emodepside, monepantel and nitazoxanide.

Hence, from our initial assessment we selected 4 compounds, which fulfilled further progression criteria (no cross resistance to already available drugs, excellent activity and toxicity profile). These four drugs are already marketed for either human (nitazoxanide, tribendimidine) or veterinary (the depsipeptide, emodepside and the aminoacetonitrile, monepantel) use. We did not compile a dossier on tribendimidine, which is registered for human use in China and is being developed for regulatory approval by a consortium composed of XPC China, Swiss Tropical and Public Health Institute (Swiss TPH), and Institute for One World Health (iOWH.) The structures of emodepside, monepantel and nitazoxanide are depicted in Figure 1.

### Drug profiles of emodepside, monepantel and nitazoxanide

Information was available through documentation in the EMA and FDA registration files (EPAR and FOI summaries, respectively) and from available Material Safety Data Sheets as well as scientific publications. Complete dossiers with these data are provided as supplementary data and a brief summary of each of the drugs is provided below. The physico-chemical characteristics of these compounds are summarized in Appendix S3.

### Emodepside

Emodepside is a semi-synthetic derivative of the cyclooctadepsipeptide PF1022A, a natural product compound produced by fermentation of the fungus *Mycelia sterilia* [36]. Anthelmintic activities of emodepside have been demonstrated in several *in vitro* and *in vivo* studies against various nematodes [37], [38]. Bayer Animal Health developed emodepside for use in cats and registered a topically administered product in combination with praziquantel to treat hookworms and ascarids (emodepside) and tapeworms (praziquantel) in Europe in 2005 and in the U.S. in 2007. The compound has a unique dual mechanism of action at the neuromuscular junction that involves on the one hand binding to a presynaptic latrophilin-like receptor and on the other hand pre- and post-synaptic interactions with a Ca^2+^-activated K^+^ ion channel (SLO-1). Binding of emodepside to the latrophilin receptor and the SLO-1 ion channel in the parasite leads to inhibition of pharyngeal pumping, paralysis and death [39], [40]. Emodepside appears to be of low general toxicity and exhibits no genotoxic properties. Although some adverse effects were noted in embryotoxicity/teratogenicity studies in rats and rabbits, the use of the compound in pregnant cats has not been associated with any teratogenic findings. This compound represents a new class of anthelmintic, which could potentially be useful to treat helminthiasis in humans. The primary issue will be cost and to our knowledge, no studies have been published to date on the efficacy of the drug against specific soil-transmitted helminths (For detailed information see Appendix 4). In addition, some safety aspects (notably safety pharmacology, reproductive toxicity and neurotoxicity) remain to be elucidated. Of note, PF1022A, the emodepside precursor should also be considered, since the drug has a broad spectrum of activity [38], [41] and might have lower production costs.

### Monepantel

The amino-acetonitrile derivatives (AAD) represent a novel class of anthelmintic drugs developed by Novartis Animal Health for use in sheep and potentially in cattle [42]. Monepantel, a member of this family, was registered in New Zealand as Zolvix® in 2009 for sheep abomasal parasites (*Haemonchus contortus*, *Trichostrongylus colubriformis, and Teladorsagia circumcincta*) and certain intestinal parasites (*Oesphagostomum* spp., *Nematodirus* spp., and *Chabertia* spp. but not *T. ovis*). As of January 2011 the product has been registered in a total of 32 countries in Australasia, Europe and Latin America. It is an agonist of a helminth-specific subfamily (DEG-3) of the nicotinic acetylcholine receptor, specifically attacking its subunit Hco-MPTL-1. Activation of the receptor through this agonist action causes hyper-contraction of the parasite body and spasmodic contraction of the pharynx [43]. It has been reported to be effective against veterinary parasites resistant to known anthelmintics including macrocylic lactones, benzimidazoles and levamisole [43], a characteristic which could potentially give it an advantage as a human health drug. As the product was just recently launched there has been no field resistance reported as yet. It has recently been demonstrated that monepantel is not active against *Strongyloides ratti* in vitro, which lacks such a MPTL-1 homolog [44]. A complete program of safety pharmacology and toxicology studies has been conducted. The compound appears to be of adequate safety with only adaptive effects noted in general toxicity studies and with no adverse effects in reproductive toxicity (paternal and embryo-foetal) observed in different animal species [45], [46]. Monepantel is also without mutagenic activity [47]. Hence, in conclusion, monepantel (i) has a clean safety profile and is reportedly not contraindicated in pregnancy; (ii) is not cross-resistant with the BZ family; and (iii) has a contemporary state-of-the-art regulatory dossier for veterinary use. The efficacy of the drug against some of the soil-transmitted helminths is not yet known. Specifically, there is no information on its activity on *Necator americanus* and *Trichuris trichiura*. Since the genomes of these two species are not published yet, and since predicting sensitivity on the basis of genomic information (such as whether the receptors conferring sensitivity to monepantel are present in *Trichuris*) might be inaccurate or insufficient, *in vitro* and *in vivo* experiments will be needed to complete its efficacy profile. Hence, we have started *in vitro* and *in vivo* studies with monepantel against *T. muris* and hookworms in our laboratories. For detailed information on monepantel see Appendix S5.

### Nitazoxanide

Nitazoxanide is an antiprotozoal drug used for the treatment of infections with *Cryptosporidium parvum* and *Giardia intestinalis*. The drug (trade name: Alinia®) is commonly given in six divided doses (500 mg bid for 3 days for adults and 200 mg bid for 3 days for children aged 4–11 years). The safety and tolerability of nitazoxanide in humans has been documented by >10 years of commercial use, during which more than 20 million people have been treated with this drug (Romark Laboratories, pers. commun.), most of them for relatively short durations ranging from 3 to 10 days. Three studies carried out in Mexico have shown that nitazoxanide achieved high cure rates against *T. trichiura* and *A. lumbricoides* [48]–[50]. For example, in Mexico cure rates of 78 and 56% were achieved against light and moderate infections with *T. trichiura* [48]. However, studies against hookworms and using single doses remain to be done. Its mode of action involves inhibition of enzymes relevant for the survival of the parasites in an anaerobic environment, such as pyruvate:ferredoxin/flavodoxin oxidoreductases, nitroreductases and/or protein disulphide isomerases. Nitazoxanide appears to be a drug with no major safety issues emerging from non-clinical safety pharmacology and toxicology studies. Specifically, reproductive toxicity was not significantly affected due to the low absorption from the gastrointestinal tract, thus allowing its use in pregnancy. Although the haematotoxicity observed in rats and dogs might warrant special consideration for the use of nitazoxanide in G6PD-deficient patients, there was no evidence from the post-marketing experience for any major safety problems associated with the human use of nitazoxanide at recommended doses. For detailed information see Appendix S6.

## Discussion

The main objective of this work was to identify potential drug candidates that would be eligible for rapid transitioning into development for human STH infections. We have not considered possible drug combinations in the present work, as this strategy has already been discussed in recent reviews [7].

Several elements have to be taken into consideration when deciding whether a compound deserves further investigation and before investment is made to provide sufficient data for an informed development track decision. Examples are: cost of goods, suitability of formulation for human use, additional non-clinical pharmacology data such as efficacy against the target human helminths, safety, including potential drug∶drug interactions, and pharmacology. Many of these issues have yet to be addressed in detail and may result in further reduction in the already sparse list of candidates.

It is clear from these searches that the majority of compounds that could be developed still come from animal health. While this analysis has focussed on single drug candidates, it is important to recognise that there may not be one simple solution to the problem, especially since humans, like their animal counterparts, may be infected with several species of helminth at one time. Thus drugs may be identified that, taken together in combination, may also enhance efficacy and also reduce the risk of generating resistance. This strategy is followed in the chemotherapy of HIV, tuberculosis and malaria, and there is no reason to suppose that it would not also be effective for STHs. Indeed, for example a recent study has shown that a combination of mebendazole and ivermectin has enhanced efficacy against trichuriasis, while protecting from the poor efficacy of ivermectin against hookworm [51].

Although drugs registered for animal health could be rapidly transitioned into humans, it is essential to conduct discussions with the relevant regulatory authorities to ensure that all the necessary pre-clinical studies have been or can be conducted to permit human studies. The examination of data compiled in analyses such as presented here, together with expertise on regulatory process, should enable to identify the most suitable candidates, requiring the lowest progression investment. While this can accelerate the transition into humans, it should also be recognised that the most expensive phase of development is yet to be faced, and not all potential candidates will make it through human efficacy testing.

Finally, it is one thing to register a drug (difficult though it may be), but getting it into use is a challenge of a different level of complexity, especially in the case of STH or other helminthic diseases. Here, the product is not chosen by the individual customer or prescriber, but is rather selected by control programmes or even internationally for procurement and distribution through MDA. With all their limitations, displacing the current BZs will be complex even for a very good drug. After all, BZs are capable of reducing infection intensity (and thus morbidity), are generally safe, are given in a single dose and the same dose for all, and are donated to a large extent. Cost-effectiveness will be an issue and will include consideration of the cost of changing policy as well, against the prospective advantages of the new drug.

We believe it was important both to conduct this analysis and to share and make the results publicly available. STH and helminthiasis in general are among the most neglected diseases in terms of drug R&D, even compared to other tropical diseases, such as those caused by kinetoplastids (leishmaniasis, African trypanosomiasis, Chagas disease) or malaria [52]. Today there are few dedicated funds for anthelmintic, particularly STH, R&D for human use; there are some potential but scattered initiatives and little or no cohesive approach thus far. With scarce resources, and the high costs and long development times for new drugs, developing the wrong candidate or a “me-too” drug (drugs that will offer no significant public health advantage over existing interventions) is not an option.

Making the results of this search publicly available will hopefully assist decision making for R&D in the community of developers and funders. However, this will only be the beginning, as more needs to be done. Based on our assessment, we will endeavour to access proprietary information through confidentiality agreements with the respective companies and to generate the data that we feel will be required to make a development track decision.

Recently, TDR, BIO Ventures for Global Health (BVGH) and the Sabin Vaccine Institute initiated discussions with a number of public and not-for-profit organizations potentially interested in drug development for helminths including developers, researchers and funding agencies. The objective is to favour an enabling environment for anthelmintic R&D, consistency and openness (with sharing of information). Hopefully a degree of cohesion can be reached. In the case of STH (and helminths at large) R&D, the situation is such (little resources, few candidate drugs, few development partners) that it must be approached considering the global R&D pipeline rather than individual initiatives by single organizations. This will hopefully provide consistency and consolidate development efforts. And this is the spirit underlying this paper.

## Supporting Information

Appendix S1 **Proposed Target Product Profile for drugs for STH.**
(DOC)

Appendix S2 **Additional details on approved animal health compounds identified, including compound class, generic name, chemical structure, current supplier, patent approval, U.S. approval, mode of action, more specifics on parasite claims and efficacy/resistance, dose rates, more on safety and toxicity issues and an overall assessment of current use in veterinary medicine.**
(XLS)

Appendix S3 **Physico-chemical data for emodepside, monepantel and nitazoxanide.**
(DOC)

Appendix S4 **Detailed information on emodepside.**
(DOC)

Appendix S5 **Detailed information on monepantel.**
(DOC)

Appendix S6 **Detailed information on nitazoxanide.**
(DOC)
