# Supplementary material for: Potential Drug Development Candidates for Human Soil-Transmitted Helminthiases
Source: PLoS Negl Trop Dis. 2011 Jun 7;5(6):e1138. doi: 10.1371/journal.pntd.0001138 (PMC3111745; doi:10.1371/journal.pntd.0001138)
Supplement: Appendix S1 — Proposed Target Product Profile for drugs for STH. (DOC) [file pntd.0001138.s001.doc]

**Target Product Profile for Soil Transmitted Helminths (STH).**

Following the identification of active compounds *in vitro* and *in vivo* during the drug development process, they may be progressed through late stage preclinical development and eventually into the clinic. The initial stages of drug development are driven by product characteristics of drugability and the ability to affect particular, probably essential, targets in the parasite(s). The Target Product Profile establishes the minimal, desirable and added value characteristics for a new compound for use in a particular parasitic disease, and are driven backwards from the potential end use (either individual treatment or public health intervention) towards the preclinical stages.

However, it should be understood that, for any new compound, not all of the characteristics might be achievable in a single step, nor is all the evidence in place at the onset. Information on items such as formulation stability do not become available until the later stages of drug development, although there may be pointers available earlier. Thus, those that are considered as **essential** characteristics are indicated in bold in the first column. Additionally, desirable characteristics are also indicated in this column in normal type. Essentiality should be viewed with caution under certain circumstances, such as where there are several conflicting indications. It should also be recognised that for a particular compound the characteristics of several different anti-parasite drug profiles might be fulfilled. The second column provides a list of additional characteristics that would provide added benefit to the product, but are less likely to be achieved.

Since it is now considered that single drugs may not provide the ideal profile by themselves, combinations of existing and/or new compounds may be needed to achieve the desired profile. The same Target Product Profiles would be expected for combinations with the added proviso that the drugs being combined do not interact in a detrimental manner pharmacokinetically and that their use does not significantly increase the likelihood of adverse reactions clinically.

Target product profiles are not intended to be proscriptive, but only act as a guide to the characteristics of an optimal drug for the particular indication as seen from the expected end use. Furthermore, they should be seen as living documents that evolve as additional information is obtained or priorities for implementation within public health activities alter. Once candidate drugs are identified for particular parasitic diseases, specific Target Product Profiles should be developed, based on these templates, which reflect what is known and/or expected of the compound.

|  | **Desirable (Minimal) Product Characteristics** | **Added Value Product Characteristics** |
| --- | --- | --- |
| **Route of administration** | **Oral (essential)** |  |
| **Activity against all species of human STN** | Active at least against adult stages of principal geohelminths, including *Ascaris*, hookworms (*Ancylostoma and Necator*), *Trichuris*, and *Enterobius* at the target dose.  Absorption minimal or significant enterohepatic recirculation of active compound after metabolism. | Additionally active against *Strongyloides,* cestodes and/or trematodes in multiple doses (<=3 days) or at higher single doses  Active against systemic helminth species with short courses of treatment (1-3 days) |
| **Activity against all stages in humans** | Active against lumen dwelling adults.  Active against ova or egg production (stop transmission) adults. | Active against migrating larvae and tissue stages of STNs |
| **Active against resistant organisms** | Novel molecule (dissimilar to existing compounds for geohelminths – neuromuscular blockers or tubulin antagonists) |  |
| **Dosing schedule** | **Single dose (preferable) or maximum two doses in one day (essential) against main STNs (*Ascaris*, hookworms, *Trichuris*)** |  |
| **Clinical safety** | Safety profile includes long term safety and mild side effects (not worse than existing agents), side effects in uninfected individuals minimal | Safety profile that permits use in control programmes |
| **Clinical efficacy** | **High efficacy: >90% cure (>95% egg reduction) of *Ascaris* and both Hookworm infections; >80% cure (>90% egg reduction) of Trichuris infections** |  |
| **Clinical use** | Safe for administration without medical supervision – especially if given without screening.  Drug with low potential for inducing resistance – novel mode of action |  |
| **Compatibility with potential partner drugs** | Concomitant treatment with ivermectin, praziquantel or benzimidazole anthelminthics. Possible to partner to improve spectrum or cure rates or reduce risk of resistance. | Capable of integration into multiple drug treatment programmes |
| **Drug-drug Interactions** | No interaction with ivermectin, benzimidazoles, or drugs for malaria or HIV/AIDS |  |
| **Use in pregnant/lactating women** | Safe during trimester 2 and 3 of pregnancy and during lactation  No teratogenetic signals in toxicology | Safe during all trimesters of pregnancy |
| **Use in infants and children** | Safe for use **from 1 year of age;**  Specific oral tablet **or suspension** for small children | Simple dosing schedule based on weight or other physical parameter |
| **Cost per treatment** | **Affordable at point of use**, therefore equivalent to cost of current treatments |  |
| **Quality** | GMP quality | Process capable of transfer to provide endemic country sourcing |
| **Storage requirements** | Two years shelf life at high humidity and temperatures (tropical standard – (40^o^ C/75%RH) | Shelf life > 2 years (extended real time) |
| **Other characteristics** |  | Also active against other helminth diseases – onchocerciasis, lymphatic filariasis and schistosomiasis.  Can administer one standard dosage for adults and children, or use height (not weight) as a determinant of dosage in children |
