# Supplementary material for: Potential Drug Development Candidates for Human Soil-Transmitted Helminthiases
Source: PLoS Negl Trop Dis. 2011 Jun 7;5(6):e1138. doi: 10.1371/journal.pntd.0001138 (PMC3111745; doi:10.1371/journal.pntd.0001138)
Supplement: Appendix S3 — Physico-chemical data for emodepside, monepantel and nitazoxanide. (DOC) [file pntd.0001138.s003.doc]

**Compilation of**

**Physico-Chemical Characteristics for**

**Emodepside, Monepantel and Nitazoxanide**

**General Information**

The physico-chemical characteristics of the three Active Pharmaceutical Ingredients (API) emodepside, monepante and nitazoxanide, considered for the potential use in anthelminthic therapy in humans, have been compiled from the respective documentation in the EMA and FDA registration files and from available Material Safety Data Sheets.

**Emodepside**

IUPAC Name: Cyclo[D-2-hydroxypropanoyl-N-methyly-L-leucyl-3-[4-(4-morpholinyl)-phenyl]-D-2-hydroxypropanoyl-N-methyl-L-leucyl-D-2-hydroxypropanoyl-N-methyl-L-leucyl-3-[4-(4-morpholinyl)-phenyl]-D-2-hydroxypropanoyl-N-methyl-L-leucyl]

CAS number: 155030-63-0

Synonyms and abbreviations: BAY 44-4400, PF1022-221, FR156742,

Molecular formula: C_60_H_90_N_6_O_14_

Molecular weight: 1119.42

Appearance: White to yellowish, odourless powder

Solubility in water: pH4: 8.1 mg/l; pH7: 5.2 mg/l; pH10: 6.1 mg/l

Solubility in organic solvents (23°C): Acetonitrile 48 %w/w; Ethanol 4%w/w;

Methanol 1%w/w

Octanol:water partition coefficient: log Pow = 4.9 (pH 7)

Rotation: -93° to -103°

Chirality: 8 chiral centres

Polymorphism: Exhibits polymorphism

Stability of Profender tablets: Stable for 36 months, with no storage temperature restrictions

**Monepantel**

INN Name: Monepantel

IUPAC Name : N-[(1S)-1-Cyano-2-(5-cyano-2-trifluoromethyl-phenoxy)-1-methyl-ethyl]-4-trifluoromethylsulfanyl-benzamide

CAS Number: 887148-69-8

Molecular Formula: C_20_H_13_F_6_N_3_O_2_S

Molecular Weight:: 473.39

Appearance: White powder

Solubility in water: 0.08 mg/l at 20°C

Solubility in organic solvents: dichlormethane: 175 g/l; ethanol: 60.7 g/l; n‑octanol: 7.3 g/l; propylene glycol: 6.9 g/l; polyethylene glycol: 156.1 g/l

Octanol:water partition coefficient: log P_ow_ = 4.7 (QSAR estimate)

Melting range: 142 -149°C (B form)

Chirality: The active substance has one chiral centre and possesses two enantiomers: the active (S)-enantiomer (parent compound A) and the inactive (R)-enantiomer which is considered an impurity.

Polymorphism: Two polymorphic forms. Form B is the thermodynamically stable form at room temperature.

Stability of API: 36 months with no special precautions for storage

Stability of Zolvix formulation: 24 to 36 months with no special precautions for storage according to nature of packaging, 12 months after opening

**Nitazoxanide**

INN Name: Nitazoxanide

IUPAC Name: 2-Acetyloxy-N-(5-nitro-2-thiazolyl)benzamide

CAS Number: 55981-09-4

Molecular Formula: C_12_H_9_N_3_O_5_S

Molecular Weight: 307.29

Appearance: Light yellow, crystalline powder

Melting Point : 202°C

Solubility in water: Practically insoluble

Solubility in organic solvents: Freely soluble in dimethyl formamide,

sparingly soluble in acetone.
