# Supplementary material for: Potential Drug Development Candidates for Human Soil-Transmitted Helminthiases
Source: PLoS Negl Trop Dis. 2011 Jun 7;5(6):e1138. doi: 10.1371/journal.pntd.0001138 (PMC3111745; doi:10.1371/journal.pntd.0001138)
Supplement: Appendix S4 — Detailed information on emodepside. (DOC) [file pntd.0001138.s004.doc]

**Summary Report on**

**Emodepside (vet: Profender®)**

**General Information**

Emodepside (CAS No. 155030-63-0; molecular weight: 1119.42) belongs to the group of cyclooctadepsipeptides, natural products, consisting of four alternating residues of N-methyl-L-leucine, two residues of D-lactate and two residues of D-phenyllactate. It is a semi-synthetic compound, the starting material for the chemical synthesis of which is produced by fermentation of the fungus *Mycelia sterilia*. Its chemical name (IUPAC) is Cyclo[D-2-hydroxypropanoyl-N-methyly-L-leucyl-3-[4-(4-morpholinyl)-phenyl]-D-2-hydroxypropanoyl-N-methyl-L-leucyl-D-2-hydroxypropanoyl-N-methyl-L-leucyl-3-[4-(4-morpholinyl)-phenyl]-D-2-hydroxypropanoyl-N-methyl-L-leucyl], and its structure is given below.


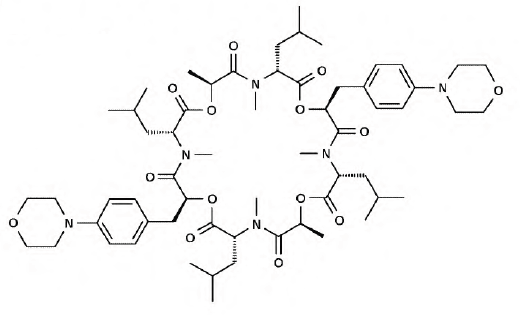
It has been approved in combination with praziquantel (trade name: Profender) for veterinary use against a variety of nematode and cestode species in the US and in Europe as a spot-on formulation for the treatment of cats and as a slow release tablet for the treatment of dogs (EMEA, 2005; FDA, 2007). A precursor of emodepside with the code name PF1022A, lacking the two morpholinyl side chains was already patented in 1990 by Meiji Seika Kaisha, and a further patent application for cyclic depsipeptides, including emodepside, was then filed in 1993 by Fujisawa Pharmaceutical Co., Ltd (Japan). Subsequently, emodepside was developed by Bayer under the code BAY 44-4400 as a veterinary product.

The first observations of anthelminthic activities in this class of compounds came from several *in vitro* and *in vivo* studies with PF1022A. Effectiveness was described in experiments against *Ascaridia galli* in chicken, against *Toxocara canis* and *T. cati* in dogs and cats, and against *Haemonchus contortus, Ostertagia ostertagi and Trichostrongylus colubriformis* in jirds, a desert rodent related to the gerbil, after oral application of low doses (up to 2 mg/kg). Higher doses of PF1022A were needed against *Heligmosomoides polygyrus* and *Heterakis spumosa* in mice (up to 50 mg/kg). No activity was observed, however, against *Trichinella spiralis* muscle stages or the tapeworm *Hymenolepis nana* (Harder et al., 2002).

In the justification for the combination of emodepside and praziquantel in its application to dogs cited in the EPAR for Profender (EMA, 2005) the following statements appear:

- *In addition to their potential to cause illness in the dog, a number of dog helminths are important zoonotic pathogens and as such are a potential hazard to public health.*
- *The test product has a broad spectrum of activity, with claimed efficacy against the common nematodes and cestodes of the dog. Based on pivotal study data (non-interference studies) presented with the application,* ***it has been confirmed that emodepside is solely responsible for effect against nematodes and praziquantel is solely responsible for effect against cestodes****. In addition, it has been shown that neither product interferes with the activity of the other.* (Emphasis added)
- *Emodepside has not been used previously for the treatment of helminth infections in the dog and has a novel mode of action. Its introduction broadens the spectrum of anthelminthics available for dog, therefore decreasing reliance on anthelminthics currently in use.*
- *The tolerance to the constituent active substances, when administered in combination, has been investigated extensively in the target species.*

Published information on the properties of emodepside is restricted mainly to investigations into its mode of action and to efficacy in animal models. Safety-related, as well as pharmacokinetic data are exclusively summarized and reviewed in the approval documentation for Profender in the EMA and FDA files (EMA, 2005; FDA, 2007).

Emodepside has not been used in humans till now, and a very recent review on anthelminthic therapies concluded that “*It is not clear at this moment if these compounds will find a place in human therapeutics.*” (van den Enden, 2009). On the other hand, the US patent awarded to Fujisawa Pharmaceutical Company for the production and use of cyclic depsipeptides, including emodepside, states the following:

“*The compound or its salt of the present invention has excellent parasiticidal activities as an anthelminthic agent for animals and human bodies. … It is also effective to parasites which infect human beings. The most common parasites in the alimentary canal of human beings are as follows: Ancylostoma genus, Necator genus, Ascaris genus, Strongyloides genus, Tichinell genus, Capillaria genus, Trichuris genus, and Enterobius genus. It is also active for other medically important parasites, which is found in the blood or other organisms or organs outside of the alimentary canal, such as Wuchereria genus, Brugia genus, Onchocerca genus and Loa genus in Filariidae, as well as parasites such as Dracunculus genus in Dracunculidae. It is also active for parasites such as Strongyloides genus and Trichinella genus in the intestinal tract in a particular conditioned parasitism outside of intestinal tract.*” (US Patent, 1996)

Although these claims are not supported by accessible scientific information and may just represent a “coverage of future interest”, they certainly point, in combination with the nematode-specific mode of action, to a possible usefulness in human therapy.

**Emodepside Mode of Action**

Emodepside has been shown to exhibit pleiotropic effects on the behaviour of the model genetic nematode *Caenorhabditis elegans*: it inhibits locomotion, feeding, egg-laying and slows development. These findings suggest that emodepside interferes with signalling at the neuromuscular junction on the body-wall muscles, pharynx and egg-laying muscles and thus inhibits three important physiological functions: locomotion, feeding and reproduction (Bull et al., 2007).

In *Ascaris suum* both cyclooctadepsipeptides, PF1022A and emodepside, were shown to lead to paralysis indicating a neuropharmacological action of these compounds. In a first study, the action of emodepside on the body wall muscle of *Ascaris suum* was found to cause (i) muscle relaxation, (ii) inhibition of muscle contraction elicited by acetylcholine (ACh) and (iii) a rapid relaxation of muscle tonically contracted by ACh. The inhibitory action of emodepside on the response to ACh was not observed in a denervated muscle strip, indicating a pre-synaptic location of its action, and electrophysiological studies showed that emodepside elicited a Ca^2+^-dependent hyperpolarization of muscle cells (Wilson et al., 2003). It was suggested that this pre-synaptic point of action might reside in a family of heptahelical transmembrane receptors, termed depsiphilins. In the parasitic nematode *Haemonchus contortus*, such a heptahelical transmembrane 110 kDa-receptor, termed HC110‑R, was identified and found to be similar to the mammalian G-protein coupled receptor latrophilin (Saeger et al., 2001; Harder et al., 2003). Binding of emodepside to this latrophilin receptor in nematodes was shown to influence presynaptic signal transduction via activation of Gqα protein and phospholipase-Cβ resulting in the mobilization of diacylglycerol, which in turn leads to activation of UNC-13 and synaptobrevin, two proteins which play an important role in presynaptic vesicle-functioning. This finally leads to the release of a neurotransmitter, exerting its effects at the postsynaptic membrane and inducing a flaccid paralysis of the pharynx and the somatic musculature in nematodes (Harder et al., 2005). It was suggested later that out of 11 different FMRFamide-like neuropeptides AF1, AF10 and PF2 could be demonstrated to have reasonable binding characteristics to the aminoterminal region of this receptor and thus to represent putative natural ligands. As it had been shown earlier by Harder et al. (2005) by electrophysiological experiments that emodepside inhibits pharyngeal pumping of the nematodes in a concentration-dependent way with an IC_50_ value of about 4 nM, the involvement of these neuropeptides in the action of emodepside may be plausible, since the neuropeptides AF1 and PF2 are known to be involved, though differently, in the control of the same process, namely pharyngeal pumping in *Caenorhabditis elegans* and *Ascaris suum*. AF1 shows stimulating effects on the frequency of pharyngeal action potential in C elegans, but inhibits the pharyngeal pumping of A. suum. However, PF2 acts inhibitory on the pharynx of both nematode species. (Mühlfeld et al., 2009).

The anthelminthic activity of emodepside was, however, shown later not to be fully explainable by its action on this receptor. Functional null mutants of *Caenorhabditis elegans* for the latrophilin gene *lat-1*, while being less sensitive to the effect of emodepside on the pharynx, they remained sensitive to the inhibitory effects of emodepside on locomotion. It was then shown that this was not due to a functional redundancy between two C. elegans latrophilins, as the double null mutant for *lat-l* and *lat-2* also remained sensitive to the effects of emodepside on locomotion. Therefore, emodepside was considered to have also latrophilin-independent effects. Mutants of *slo-1*, which encodes a Ca^2+^-activated K^+^ channel were highly resistant to the inhibitory effect of emodepside on both pharyngeal and locomotor activity. Tissue-specific genetic rescue experiments revealed that emodepside acts through SLO-1 expressed in either body wall muscle or in neurones to inhibit locomotion. In contrast, in the pharyngeal system, emodepside acts through SLO-1 in neurones, but not muscle, to inhibit feeding. These data suggested then that emodepside causes inhibition of feeding via a neuronal SLO‑1-dependent pathway which is facilitated by LAT-1 whilst it signals through a latrophilin-independent, SLO-1-dependent pathway, in either neurones or body wall muscle, to inhibit locomotion. (Guest et al., 2007). A model for this dual action has been presented by Holden-Dye et al. (2007; see figure 1, overleaf).

Figure 1: Proposed emodepside action at the neuromuscular junction (from Holden-Dye et al., 2007)

Finally, in order to account for the variability of the effect of emodepside on the different life stages of helminths, the transcript levels of the latrophilin-like receptor depsiphilin in *Ancylostoma caninum* were analyzed in eggs, LI, L3, male and female adult worms using quantitative real-time PCR. Depsiphilin was found to be transcribed in all five examined stages, but a significantly lower transcript level was observed in third-stage larvae. The authors concluded that a correlation between these findings and a reduced emodepside activity would remain to be investigated (Krüger et al., 2009).

**Non-Clinical Efficacy Data**

Extensive efficacy data have been provided in the submission documentation for the registration of Profender in Europe and the USA. Since the veterinary product Profender is a combination of emodepside and praziquantel, only part of these data were obtained on the individual components, with most of the information concerning the combination. Furthermore, a limited number of publications on the non-clinical efficacy of emodepside are also available. It has to be recognized, however, that all the studies – save only a few exceptions – conducted with emodepside, whether contained in the submission files or available in the open literature, are directed to the helminth species important to the veterinary target species, specifically nematodes infecting cats and dogs. There are no efficacy data on helminth species that may be important pathogens for humans.

With this limitation in mind, the following can be stated with respect to non-clinical efficacy data.

In Europe, the approved indications specify

- for cats: “Roundworms (Nematodes): *Toxocara cati* (mature adult, immature adult, L4 and L3), *Toxascaris leonina* (mature adult, immature adult and L4), *Ancylostoma tubaeforme* (mature adult, immature adult and L4); Tapeworms (Cestodes): *Dipylidium caninum* (adult), *Taenia taeniaeformis* (adult), *Echinococcus multilocularis* (adult)”;
- and for dogs: “Roundworms (Nematodes): *Toxocara canis* (mature adult, immature adult, L4 and L3), *Toxascaris leonina* (mature adult, immature adult and L4), *Ancylostoma caninum* (mature adult and immature adult), *Uncinaria stenocephala* (mature adult and immature adult), *Trichuris vulpis* (mature adult, immature adult); Tapeworms (Cestodes): *Dipylidium caninum*, *Taenia spp*., *Echinococcus multilocularis* (mature adult and immature), *Echinococcus granulosus* (mature adult and immature)”.

In the US Profender topical solution is indicated “for the treatment and control of hookworm infections caused by *Ancylostoma tubaeforme* (adults, immature adults, and fourth stage larvae), roundworm infections caused by *Toxocara cati* (adults and fourth stage larvae), and tapeworm infections caused by *Dipylidium caninum* (adults) and *Taenia taeniaeformis* (adults) in cats.”(FDA, 2007).

As it has been remarked in the General Information, the EPAR succinctly states that emodepside is exclusively responsible for the activity of Profender against nematodes. This statement is supported by the exclusive use of nematodes in the dose-range finding studies for emodepside in cats and dogs, summarized in the EPAR as follows:

“The pivotal dose determination studies (in the cat) were conducted using *Toxocara cati* and *Dipylidium caninum*. The Applicant suggested that the use of these parasites in the pivotal studies was justified on the basis that they are dose-limiting species.

A study on the efficacy of emodepside against artificial infection with *Ancylostoma sp.* in cats in South Africa concluded that emodepside was effective against *Ancylostoma braziliense* at doses of 2 and 4 mg/kg. No adverse reactions related to the application of the test products were observed during the study.

The efficacy of emodepside at various doses against natural infection with *Ancylostoma spp*. and *Toxocara spp*. in cats in Australia was conducted with a GCP-compliant study. The results demonstrated that 2 mg emodepside/kg was highly effective against naturally acquired infections of *Ancylostoma spp*. and/or *Toxocara spp*. No adverse events attributable to treatment were observed.

A series of pilot studies using development formulae were conducted to identify an effective dose of emodepside in the dog. The target species investigated were *Uncinaria stenocephala*, *Ancylostoma caninum* and *Trichuris vulpis*. In this series of studies, emodepside, when administered at a dose of 0.5 mg/kg had acceptable efficacy against *U. stenocephala* and *A. caninum*. However, this dose was only 73.7% effective against *T. vulpis*.

Based on these data, 1.0 mg emodepside was chosen as the minimum effective dose for further studies and *T. vulpis* was considered a dose limiting species.

Efficacy against *T. vulpis* at a dose of 1 mg emodepside/kg was demonstrated in a further pilot study. In a pivotal GCP study, efficacy was investigated against mature and immature adult *T. vulpis*: Emodepside was 99.6% and 100% effective against immature adult and adult *T. vulpis* respectively at a dose of 0.5 mg/kg. While 0.5 mg emodepside/kg was shown to be effective in the pivotal study, the Committee could accept the decision of the Applicant to choose 1.0 mg emodepside as the minimum effective dose based on the findings of earlier studies.” (EMA, 2005).

Additionally, the efficacy summary for emodepside as an individual active ingredient in the FDA review cites studies on nematode species in cats as follows: “The effectiveness of 1, 2, and 4 mg of emodepside per kg body weight was tested against *Toxocara cati* and/or *Ancylostoma species* (spp.). Two studies showed that 1 mg/kg emodepside was > 90% effective against *Ancylostoma spp*. but a third showed that dose only 51% effective against *Ancylostoma braziliense*. Another study showed that 1 mg/kg was 94.6% effective against *Ancylostoma spp*. but only 42% effective against *T. cati*. These studies showed that the appropriate dose of emodepside against *T. cati* was between 1 and 2 mg/kg but with variable consistency. A dose of 3 mg/kg was chosen to ensure consistency.” (FDA, 2007).

The activity of emodepside was studied against larval and adult stages of three rodent nematodes. While emodepside was shown to act strongly against the adult stages of the rat nematodes *Nippostrongylus brasiliensis* and *Strongyloides ratti*, as well as against the mouse nematode *Heligmosomoides polygyrus*, its actions against the larval stages of these nematodes did vary according to the species. High efficacy against the lung and intestine larval stages of *N. brasiliensis* and *S. ratti* were contrasted to an only partial effectiveness of higher emodepside dosages against the larval stages of *H. polygyrus* in the intestine (Harder and von Samson-Himmelstjerna, 2001). Treatment with the spot-on solution of the emodepside-praziquantel combination of pet reptiles (snakes, turtles, geckos, lizards, etc.), infected with a variety of nematodes that were identified by microscopic faecal examination as belonging to the families Oxyuridae, Ascaridae, Strongylidae, Trichostrongylidae and Capillaridae, resulted in complete disappearance of the nematodes already one day after treatment (Mehlhorn et al., 2005).

The effects of emodepside on the egg and free-living larval stages of several species of gastrointestinal nematodes were examined in *in vitro* assays addressing its possible inhibitory effects on egg hatching, larval development and larval motility. The egg hatch assay (EHA) and the larval development assay (LDA) were carried out using stages of *Haemonchus contortus* and cyathostomins of the horse. The larval motility inhibition test (LMIT) was performed with larval stages from *Cooperia oncophora, Ostertagia ostertagi, H. contortus, Ancylostoma caninum* and cyathostomins. Levamisole and ivermectin were included as comparators in this study. In the EHA no ovicidal effects of emodepside were observed even at the highest concentrations possible. The LDA showed the inhibitory activity of emodepside on larval development, which differed only slightly between the species tested, and also the LMIT demonstrated the inhibitory effects of emodepside on larval migration, with similar EC_50_ values across all species tested. For all drugs tested a clear order of potency was observed: Ivermectin was the most, levamisole the least potent anthelminthic. With EC_50_ values of 2.25-8.28 µM, emodepside ranged between the two (Schürmann et al., 2007).

Finally, a series of publications describes the efficacy of emodepside in the combination with praziquantel against mature adult, immature adult and larval stages of *Toxocara canis* and *Toxascaris leonina*, mature and immature adult hookworms (*Ancylostoma caninum* and *Uncinaria stenocephala*), and against mature and immature adult whipworms (*Trichuris vulpis*) in dogs. In all studies, efficacies of >90% were observed against all species and their development stages investigated (Altreuther et al., 2009; Schimmel et al., 2009a; Schimmel et al., 2009b). Most publications on the efficacy of emodepside in the veterinary product Profender are concerned, however, with the efficacy of the spot-on formulation on the same few parasites; consequently, they are not of importance for the use in humans and are not summarized here.

**Non-Clinical Pharmacokinetics and ADME Data**

No information on pharmacokinetics and ADME has been published in the open literature. In the EPAR on Profender, the data submitted on these properties of emodepside have been summarized as follows (EMA, 2005):

“Pharmacokinetics was studied following i.v. administration in the rat. In summary:

- approximately half the administered dose was excreted in the first 24 hours, with the remainder being excreted slowly, with up to 8 % still being detected at 168 hours,
- faecal excretion predominated with only 2-3 % of the dose being found in urine,
- the elimination half life was calculated to be 39-51 hours,
- the bioavailability was 53-57 % at both dose levels,
- the highest levels of radioactivity were found in the fat, at all dose levels and time points, which was probably acting as a depot for the absorbed radioactivity,
- unchanged emodepside was the major excretion product, accounting for 45‑56 % of the dose. There are numerous small metabolites with only 4 groups accounting for >5 % of the dose.

In addition to the pivotal ADME study in the rat, reports of other studies were provided. The principal observations were:

- In two subacute toxicity studies in rats, emodepside was administered in the food. Blood concentrations were comparable on days 9 and 28 suggesting that steady state had been reached by day 9. While there appeared to be dose proportionality between the low and medium dose group, there appeared to be some degree of saturation between the middle and high dose groups.
- When a formulation of the active substance in ethanol was applied enterally to dogs, the highest mean plasma concentrations were found in the 5 and 15 mg/kg group 1 hour after application, whereas T_max_ was reached after 0.5 hours for the 45 mg/kg group. It is noted that C_max_ increased proportionally up to 15 mg/kg. Thereafter, a saturation of absorption became apparent.”
- When emodepside was given orally to fed dogs, C_max_ was nearly twice as high compared to fasted animals. Similarly, AUC_0-12_ _h_ was markedly higher in the fed state compared to the unfed state. Effects of feeding on emodepside C_max_ and AUC_0-12 h_ were statistically significant.

More precise data are mentioned in the SmPC for Profender tablets, where it is stated that after treatment with an emodepside dose of 1.5 mg/kg mean maximum plasma concentrations of 47 μg/l were observed. Maximum concentrations were reached 2 hours after treatment and an elimination half-life of 1.4 to 1.7 hours was determined (SPC, 2008).

No information on pharmacokinetics and ADME is presented in the respective FDA summary.

**Non-Clinical Safety Data**

No toxicology data have been published in the open literature. The non-clinical safety investigations on emodepside, as reported in the EPAR, contain the results of a full toxicology programme, but no information on safety pharmacology. The FDA Review, on the other hand, does contain no toxicity information but only data on tolerance in the target species (cats and dogs). The toxicology data obtained with emodepside have been summarized in the EPAR and indicate the following properties:

Emodepside has a low acute toxicity by a variety of routes in mice and rats, with overt signs of toxicity at high doses including depressed neurological and respiratory function. Four repeat dose toxicity studies after oral administration of emodepside were conducted in rats and mice for up to 17 weeks. Results obtained in rats included abnormal clinical signs (neurological function, ataxia, increased motility, behaviour, piloerection, respiration) and adverse effects on weight gain and water/feed consumption. The liver with increased enzyme activity and reduced protein synthesis, the adrenal glands, pancreas and reproductive system were the principal target organs for toxicity, and an increase in glucagon-secreting cells with a trend towards significant hyperglycaemia, polydipsia and polyphagia was observed. It is noteworthy that many histopathological changes did not reverse during the 4-week recovery period. Although potent effects on various endocrinological end-points were identified, emodepside does not appear to interact directly with androgenic or estrogenic receptors. Studies in cats, dogs, mice and rabbits, however, showed no significant or consistent hyperglycaemia nor altered metabolism of fat and proteins or increase in food consumption. In studies in dogs, neurological effects (tremor, ataxia) were observed at high doses that were greater when dogs were fed compared to when not fed. Based on the pharmacokinetic findings it appears that these observations are linked to the higher serum concentrations of emodepside achieved when dogs are fed at the time of treatment. From these studies in rats, NOELS were determined to be 0.73-1.11 mg/kg in the subchronic and 4.4-4.6 mg/kg in the subacute studies. In mice, the NOEL was 10.5-16.8 mg/kg.

Reproductive toxicity investigations are more or less restricted to studies in rats and rabbits on embryotoxicity and teratogenicity (Segment II). No Segment I study on fertility, nor a Segment III study on reproductive performance was provided. Some adverse effects on reproductive and endocrine tissues have been observed in the repeat-dose toxicity studies conducted in rodents, however, indicating potential adverse effects on fertility. On the other hand, the use of the drug in pregnant cats was found not to be associated with any teratogenic findings, and this was considered to obviate the need for a Segment III study. In the studies conducted on embryotoxicity and teratogenicity in the rats, both the ovarian weight and the gestation rate were unaffected by treatment. Clinical signs of systemic maternal toxicity were evident at dose rates ≥ 6 mg/kg. Overall, severe maternal toxicity at 18 mg/kg resulted in adverse effects on foetal development. The NOEL for maternal toxicity was 2 mg/kg and the NOEL for developmental toxicity was 0.5 mg/kg. In rabbits, the effects were similar to the rat studies. The NOEL for developmental toxicity in the rabbit was 5 mg/kg.

A battery of appropriate *in vitro* and *in vivo* tests indicates that emodepside is a non-mutagenic substance. Although no carcinogenicity data were provided, the negative results in the mutagenicity studies indicated that such studies should not be required. Emodepside is non-irritating to the eyes and skin, and does not appear to be a skin sensitising agent.

An *in vitro* study was conducted on the interaction of emodepside with the multi-drug resistance protein (MDR). Compared to ivermectin, emodepside was found to be more effectively effluxed in MDR-1 cells and this active transport was not saturable in the concentration range tested. These data suggest that emodepside will not be able to cross membranes such as the blood brain barrier. It was concluded that there may be a potential for interaction with other P-glycoprotein dependent substrates.

**Conclusions and Recommendations**

Emodepside is a new anthelminthic with a dual mode of action at the neuromuscular junction by interacting with a presynaptic latrophilin-like receptor and pre- and post-synaptic SLO-1, a Ca^2+^-activated K^+^ ion channel. Inhibition of feeding is thus caused via a neuronal SLO‑1-dependent pathway which is facilitated by LAT-1, whilst locomotion is inhibited by signalling through SLO-1 in either neurones or body wall muscle, resulting in paralysis and death of the parasites.

Although the mode of action indicates that emodepside should be active against nematodes in general, and although claims of efficacy against nematodes important in human health situations are made, there are no specific data on the activity of emodepside against these species. Some information should thus be obtained in *in vitro* studies, especially with the view on active concentrations and their relation to levels attainable in the clinical situation.

Emodepside was found to have a low toxicity with liver, adrenal glands, pancreas and reproductive system as the principal target organs for toxicity. Adverse effects were noted in embryotoxicity/teratogenicity studies in rats and rabbits, but the use of the molecule in pregnant cats has not been associated with any teratogenic findings. Genotoxicity studies were negative, and emodepside is non-irritating to the eyes and skin, and does not appear to be a skin sensitising agent. There was no information relating to experience in humans. The non-clinical safety information seems, therefore, to be more or less complete with a few exceptions: No safety pharmacology studies have been performed, and reproductive toxicity information is incomplete. While the still missing reproductive toxicity data could be generated in a time frame up to the NDA submission, safety pharmacology data, especially the potential effects of emodepside on the cardiovascular system (QT prolongation), would have to be generated before entering first clinical trials.

The Lazar Toxicity Prediction software, which compares an unknown agent in a structure-activity relationship estimation to the maximum recommended daily doses (MDD) of pharmaceuticals in the FDA database, provides a value of 0.00789 mM/kg/day, equal to a dose of 8.8 mg/kg/day. As the confidence value placed on the calculated MDD is with 5% rather low, and in view of the low NOELs with Human Equivalent Dose values of around 0.16 – 1.4 mg/kg, the estimate may be considered as being on the high side.

One advantage of a possible use of emodepside in the context of anthelminthic treatments in disease-endemic countries would most probably be its single-dose regimen, provided the efficacy of a single dose can be reproduced and proven in humans. One drawback, however, could be the potential requirement to take the drug in a fasted state in order to minimize the possibilities for the occurrence of neurological side effects.

In conclusion, the development of emodepside for the treatment of human nematode infections should certainly be considered.

**References**

Altreuther G, Schimmel A, Schroeder I, Bach T, Charles S, Kok DJ, Kraemer F, Wolken S, Young D, Krieger KJ (2009). Efficacy of emodepside plus praziquantel tablets (Profender tablets for dogs) against mature and immature infections with *Toxocara canis* and *Toxascaris leonina* in dogs. Parasitol Res 105 Suppl 1: S1‑S8.

Bull K, Cook A, Hopper NA, Harder A, Holden-Dye L, Walker LJ (2007). Effects of the novel anthelminthic emodepside on the locomotion, egg-laying behaviour and development of *Caenorhabditis elegans*. Int J Parasitol 37: 627-636.

EMA European Medicines Agency (2005). European Public Assessment Report on Profender. Downloadable from http://www.ema.europa.eu/vetdocs/vets/Epar/profender/profender.htm

FDA Food and Drug Administration (2007). Freedom of Information Summary on Profender Topical Solution (NADA 141-275). Downloadable from: http://www.fda.gov/AnimalVeterinary/Products/ApprovedAnimalDrugProducts/FOIADrugSummaries/ucm056939.htm

Guest M, Bull K, Walker RJ, Amliwala K, O'Connor V, Harder A, Holden-Dye L, Hopper NA (2007). The calcium-activated potassium channel, SLO-1, is required for the action of the novel cyclo-octadepsipeptide anthelminthic, emodepside, in *Caenorhabditis elegans*. Int J Parasitol 37: 1577-1588.

Harder A, von Samson-Himmelstjerna G (2001) Activity of the cyclic depsipeptide emodepside (BAY 44-4400) against larval and adult stages of nematodes in rodents and the influence on worm survival. Parasitol Res 87: 924-928.

Harder A, von Samson-Himmelstjerna G (2002). Cyclooctadepsipeptides - a new class of anthelmintically active compounds. Parasitol Res (2002) 88: 481-488

Harder A, Schmitt-Wrede H-P, Krücken J, Marinovski P, Wunderlich F, Willson J, Amliwala K, Holden-Dye L, Walker R (2003). Cyclooctadepsipeptides—an anthelmintically active class of compounds exhibiting a novel mode of action. Int J Antimicrob Agents 22: 318-331

Harder A, Holden-Dye L, Walker R, Wunderlich F (2005) Mechanisms of action of emodepside. Parasitol Res 97 Suppl 1: S1-10.

Holden-Dye L, O'Connor V, Hopper NA, Walker RJ, Harder A, Bull K, Guest M (2007). SLO, SLO, quick, quick, slow: calcium-activated potassium channels as regulators of *Caenorhabditis elegans* behaviour and targets for anthelmintics. Invert Neurosci 7:199-208

Jeschke R, Iinuma K, Harder A, Schindler M, Murakami T (2005). Influence of the cyclooctadepsipeptides PF1022A and PF1022E as natural products on the design of semi-synthetic anthelmintics such as emodepside. Parasitol Res 97 Suppl 1: S11-S16.

Krüger N, Harder A, von Samson-Himmelstjerna G (2009). The Putative Cyclooctadepsipeptide Receptor Depsiphilin of the Canine Hookworm Ancylostoma caninum. Parasitol Res 105:S91-S100

Mehlhorn H, Schmahl G, Frese M, Mevissen I, Harder A, et al. (2005) Effects of a combinations of emodepside and praziquantel on parasites of reptiles and rodents. Parasitol Res 97 Suppl 1: S65-S69.

Mühlfeld S, Schmitt-Wrede H-P, Harder A, Wunderlich F (2009). FMRFamide-like neuropeptides as putative ligands of the latrophilin-like HC110-R from *Haemonchus contortus*. Mol Biochem Parasitol 164:162-164

Saeger B, Schmitt-Wrede HP, Dehnhardt M, Benten WP, Krücken J, Harder A, Von Samson-Himmelstjerna G, Wiegand H, Wunderlich F (2001). [Latrophilin-like receptor from the parasitic nematode Haemonchus contortus as target for the anthelminthic depsipeptide PF1022A.](http://www.ncbi.nlm.nih.gov/pubmed/11344131?itool=EntrezSystem2.PEntrez.Pubmed.Pubmed_ResultsPanel.Pubmed_RVDocSum&ordinalpos=2) FASEB J 15(7):1332-4.

Schimmel A, Altreuther G, Schroeder I, Charles S, Cruthers L, Ketzis J, Kok DJ, Kraemer F, McCall JW, Krieger KJ (2009a) Efficacy of emodepside plus praziquantel tablets (Profender tablets for dogs) against mature and immature adult *Ancylostoma caninum* and *Uncinaria stenocephala* infections in dogs. Parasitol Res 105 Suppl 1: S9-16.

Schimmel A, Altreuther G, Schroeder I, Charles S, Cruthers L, Kok DJ, Kraemer F, Krieger KJ (2009). Efficacy of emodepside plus praziquantel tablets (Profender tablets for dogs) against mature and immature adult *Trichuris vulpis* infections in dogs. Parasitol Res 105 Suppl 1: S17-S22.

Schürmann S, Harder A, Schnieder T, von Samson-Himmelstjerna G (2007). Effects of Emodepside on Egg Hatching, Larval Development and Larval Motility in Parasitic Nematodes. Parasitol Res 101:S45-S56

SPC Summary of Product Characteristics (2008) Profender Product Information, European Medicines Agency. Downloadable from: http://www.ema.europa.eu/vetdocs/vets/Epar/profender/profender.htm

United States Patent 5514773. Depsipeptide derivatives, production thereof and use thereof. Downloadable from: http://www.freepatentsonline.com/5514773.html

van den Enden E (2009). [Pharmacotherapy of helminth infection.](http://www.ncbi.nlm.nih.gov/pubmed/19191680?itool=EntrezSystem2.PEntrez.Pubmed.Pubmed_ResultsPanel.Pubmed_RVDocSum&ordinalpos=1) Expert Opin Pharmacother 10(3):435-451.

von Samson-Himmelstjerna G, Harder A, Sangster NC, Coles GC (2005) Efficacy of two cyclooctadepsipeptides, PF1022A and emodepside, against anthelminthic-resistant nematodes in sheep and cattle. Parasitology 130: 343-347.

Willson J, Amliwala K, Harder A, Holden-Dye L, Walker RJ (2003) The effect of the anthelminthic emodepside at the neuromuscular junction of the parasitic nematode Ascaris suum. Parasitology 126: 79-86.
