# Supplementary material for: Potential Drug Development Candidates for Human Soil-Transmitted Helminthiases
Source: PLoS Negl Trop Dis. 2011 Jun 7;5(6):e1138. doi: 10.1371/journal.pntd.0001138 (PMC3111745; doi:10.1371/journal.pntd.0001138)
Supplement: Appendix S5 — Detailed information on monepantel. (DOC) [file pntd.0001138.s005.doc]

**Summary Report on**

**Monepantel (vet: Zolvix®)**

**General Information**

Monepantel, with the chemical name (IUPAC) of N-[(1S)-1-Cyano-2-(5-cyano-2-trifluoromethyl-phenoxy)-1-methylethyl]-4-trifluoromethylsulfanylbenzamide (CAS number 887148-69-8; MW 473.39; chemical structure see below), is the first member of the very recently discovered class of amino-acetonitrile derivatives (AAD) that have been shown to possess anthelminthic activity. It is registered and marketed in the form of the pure S-enantiomer in Australia, New Zealand and Europe as an anthelminthic for veterinary use under the trade name of Zolvix®.

The US Patent Application 2006/0264653 A1, published on November 23, 2006, specifically claims efficacy against helminths in humans as follows:

Although it is stated in this patent application that monepantel may exhibit anthelminthic activities suitable for the treatment of such diseases in humans, the patent claim cannot be considered as scientific proof of these activities, since a patent application is intended to completely cover all potential uses of this new drug. On the other hand, the mode of action of this class of compounds seems to make it plausible that nematodes in general would be susceptible to the action of monepantel. Notwithstanding these claims of efficacy against human pathogens, monepantel was first developed as a veterinary drug, most probably because the activity of this class

of compounds has been discovered in such a setting (i.e., in the Novartis Centre de Recherche Santé Animale, St.Aubin, Switzerland), and possibly also because of marketing considerations. Consequently, the information about efficacy and safety available from official documents (the EMA EPAR for Zolvix of November 2009), and published literature is exclusively concerned with the effects studied and observed in laboratory and/or farm animals, especially sheep.

**Monepantel Mode of Action**

Monepantel acts differently from all other anthelminthics using a unique mode of action by attacking the nematode-specific sub-family DEG-3 of the nicotinic acetylcholine receptor (nAChR), and more specifically the sub-unit Hco-MPTL-1 (EMA, 2009; Kaminsky et al., 2008a; Rufener et al., 2009). Acetylcholine receptors are acting at the neuromuscular junction, i.e., the interface between nerve and muscle cells where nerve impulses are transmitted to muscular cells, in transmitting excitatory signals from the nerves to the muscle. Interference with the function of these receptors, such as seen with the blocking activity of curare, will lead to muscular paralysis. Worms paralyzed in this way in the intestine will therefore be unable to sustain normal biological functions that require functional neuromuscular signal transmission. AADs active against parasitic nematodes thus exerted marked effects on the movement, growth and viability of the free-living nematode *Caenorhabditis elegans*, causing hypercontraction of the body wall muscles leading to paralysis, spasmodic contractions of the anterior portion of the pharynx and ultimately death. The underlying molecular biology for this mode of action on nematodes has primarily been studied using a forward genetic screen with *C. elegans* mutants resistant to the action of AADs. Of 44 resistance alleles isolated, 36 fell into a single complementation group containing two genes, *acr-17* and *acr-23*, which encode predicted nicotinic acetylcholine receptor (nAChR) subunits, belonging to a nematode-specific subfamily of nAChRs, which does not occur in mammals. The finding that high-level (>1000-fold) resistance is the major phenotype resulting from loss of functional ACR-23 protein is most simply explained by hypothesizing that the substance is a direct agonist of ACR-23-containing ion channels. (Kaminsky et al., 2008a).

Such a specific interaction may be considered favourable on the one hand, since it should not occur in mammals where this specific subunit of the nicotinic acetylcholine receptor is not expressed and present, thus promising low toxicity and a lack of serious side effects, but on the other hand, development of resistance might become a problem in such a case, since any genetic change altering the drug binding site of the receptor subunit might abolish the drug’s activity, as the experiments with the various mutants have shown.

**Non-Clinical Efficacy Data**

The compound has a very rapid, potent and penetrant neuromuscular effect on *Caenorhabditis* *elegans* that can be translated into relevant anthelminthic activity in the *in vivo* situation.

A number of papers have been published reporting on results of dose-finding and efficacy studies conducted under field conditions.

Monepantel was demonstrated to be the most suitable amongst a number of AADs investigated, as it eliminated many tested pathogenic nematode species, both at larval and adult stages, at a dose of 2.5 mg/kg bodyweight in sheep and 5.0 mg/kg bodyweight in cattle. The same doses were sufficient to cure animals infected with resistant or multi-drug-resistant nematode isolates (Kaminsky et al., 2008b).

Dose finding studies were conducted with a variety nematode infections in sheep. Sheep were infected with fourth stage larvae (L4) of the target nematodes (selected from *Haemonchus contortus, Teladorsagia (Ostertagia) circumcincta, Teladorsagia trifurcata, Trichostrongylus axel, Trichostrongylus colubriformis, Trichostrongylus vitrinus, Cooperia curticei, Cooperia oncophora, Nematodirus battus, Nematodirus filicollis, Nematodirus spathiger, Chabertia ovina and Oesophagostomum venulosum*) and treated orally with monepantel at doses of 1.25, 2.5 or 5.0 mg/kg. Monepantel proved highly effective at 2.5 and 5.0 mg/kg, but was only moderately effective against some nematode species (L4 stage) at 1.25 mg/kg. The results also confirmed that monepantel will effectively control L4 stages of nematodes resistant to at least some of the currently available broad-spectrum anthelminthic classes (macrocyclic lactone resistant strains were not included in the studies). It was concluded that 2.5 mg/kg would be a suitable minimum dose rate for a commercial product (Hosking et al., 2008). In order to confirm the minimum therapeutic oral dose of monepantel to control fourth stage (L4) gastro-intestinal nematode larvae (target species were the same as above*)*, sheep infected with a defined selection of the target nematodes were treated with monepantel at a single dose of 2.5 mg/kg. The results demonstrated high (95 < 100%) efficacy and the broad-spectrum activity of monepantel against L4 larvae of common gastro-intestinal nematodes in sheep and its favourable safety profile was judged to represent a significant advance in the treatment of parasitic gastro-enteritis in this animal species (Hosking et al., 2009a). Further dose-finding studies were conducted for the control of adult gastro-intestinal nematodes. Sheep infected with adult stages of various nematode species were treated with monepantel doses of 1.25, 2.5 or 5.0 mg/kg. It was again concluded that 2.5 mg/kg would be a suitable dose for the control of nematode infections (Kaminsky et al., 2009).

A number of clinical field studies in sheep confirmed that monepantel, at a single oral dose of 2.5 mg/kg was an effective treatment, leading to practically complete eradication of the intestinal nematodes. Furthermore, efficacy was also against some populations resistant to the currently available broad-spectrum anthelminthics. Monepantel was well tolerated by the treated sheep, with no treatment related adverse events documented and was also safe for the human operators when used in a field situation (Hosking et al., 2009b; Sager et al., 2009; Mason et al., 2009).

Finally, an analysis of pooled data from a series of controlled studies was also reported. It showed that, for most nematode species, the pooled efficacy was greater than 99%, and for the remaining few species, efficacy was greater than 90%. These data were well supported by field studies conducted across five countries, where the pooled efficacy (on the basis of faecal worm egg count reduction) was in most cases greater than 99% (Hosking et al., 2010).

It is stated furthermore that in *in vitro* assays the major metabolite, monepantel sulfone, has shown similar potency as the parent compound, monepantel, against gastro-intestinal nematode larvae (Karadzovska et al., 2009), although no supporting data seem to have been presented to the authorities, as no such a statement is to be found in the EPAR.

Although these data do not have an immediate bearing on the human situation with nematode infections, the general occurrence and importance of the drug target in these parasites provides a certain reassurance for the possibility of finding human therapeutic uses for this substance.

**Non-Clinical Pharmacokinetics and ADME Data**

Pharmacokinetic and ADME data have been submitted to EMA for the registration of Zolvix and are summarized in the respective EPAR (EMA, 2009).

In rats the bioavailability based on ^14^C-monepantel was approximately 30 % after a single oral dose of 2.5 mg/kg, while the oral bioavailability of parent monepantel was estimated to be 9.4 % indicating that a part of the absorbed oral dose is converted to the sulfone by first-pass metabolism. Terminal elimination of radioactivity from plasma occurred with a half-life of 40 to 60 hours. Monepantel excretion was mainly via the faeces (70 to 97 %) within 3 days as unchanged parent (25 % of the mean daily dose) and the sulfone metabolite (52 % of the daily dose).

After repeated daily oral administration of 10 mg/kg for seven days, the major part of administered radioactivity was recovered in faeces (60 to 80 %) and some in urine (3 to 6 %). Total (^14^C) residue concentrations were generally highest in the liver and fat, followed by adrenals, pancreas and ovaries, low in blood and muscle and intermediate in kidney. In all tissues residues were characterized as mainly parent drug and the sulfone metabolite. In addition three minor metabolite fractions and a further five very minor fractions were detected in tissues.

*In vitro* studies using liver microsomes were performed to determine the intrinsic clearance of parent compound in mouse, rat, dog and human, with a view to monitoring a possible relationship between toxicological response and metabolic stability of parent compound in various species and to comparing the interspecies pattern and nature of metabolites. High intrinsic clearance values of monepantel (at 1 μM) were observed in liver microsomes of rat and man, whereas only moderate values were determined in liver microsomes of mouse and dog. The pattern of metabolites in incubations at 10 μM monepantel was comparable between the four species.

In another in vitro study, the plasma protein binding of monepantel to rat, dog, sheep and bovine plasma was investigated. Initial experiments confirmed the stability of monepantel in rat plasma and 30 h were required to reach equilibrium. Using equilibrium dialysis against ultrafiltrated (protein free) plasma, it was determined that protein binding was >99 % for rat and dog plasma, and 98.4 % and 97.2 % for bovine and sheep plasma, respectively (EMA, 2009).

In a publication, the pharmacokinetic properties of monepantel and its sulfone metabolite were reported in sheep following intravenous (i.v.) and oral administrations. The sulfone metabolite was rapidly formed and predominated over monepantel 4 h after dosing, irrespective of the route of administration, as it was demonstrated that approximately the same amount of monepantel sulfone was generated whether monepantel was given intravenously or orally (ratio for monepantel sulfone of oral AUC_(0-∞)_ to i.v. AUC_(0-∞)_ of 0.94). The overall bioavailability of monepantel was 31% with AUCs of 211, 671 and 1920 ng/ml•h for oral doses of 1, 3 and 10 mg/kg, respectively. Exposure to monepantel sulfone in terms of AUC was much higher with values of 3376, 11125 and 19110 ng/ml•h following the above oral doses of monepantel, respectively. The steady-state volume of distribution, total body clearance and mean residence time of monepantel were 7.4 l/kg, 1.49 l/kg•h and 4.9 h, respectively, and 31.2 l/kg, 0.28 l/kg•h and 111 h, respectively, for monepantel sulfone. Excretion of monepantel and monepantel sulfone was exclusively through the faecal route, but mass balance studies showed that a substantial part of monepantel sulfone should be further metabolized and be excreted through the kidney (Karadzovska et al., 2009).

**Non-Clinical Safety Data**

A complete programme of safety pharmacology and toxicology studies has been conducted for the submission of monepantel which is summarized in the EPAR of Zolvix®. A limited set of these toxicology data has also been published (Kaminsky et al., 2008b), and reproductive safety studies in sheep have been conducted and published (Malikides et al., 2009a; Malikides et al., 2009b; Malikides et al., 2009c).

Safety pharmacology has been investigated in rats. There was no effect on intestinal motility and stomach weight in a charcoal propulsion test, and no changes in the Irwin screen test at the oral limit dose of 2000 mg/kg in rats. Effects on cardiovascular and respiratory parameters were studied in the anaesthetised rat after the same dose applied intraduodenally. Systolic, diastolic and mean blood pressure, heart rate, electrocardiograms, respiratory rate, tidal volume and minute volume (calculated) were monitored before and for up to 4.5 hours after treatment. The effects were limited to a small decrease in mean respiratory rate and minute volume, but this change was considered not to be biologically significant. Furthermore, the observations in the toxicological and target species tolerance studies provided no evidence of a pharmacological effect. Since no specific signs were observed even in high single and repeated dose studies in the various species (mouse, rat, rabbit, dog, sheep), it is concluded that the compound is devoid of a relevant pharmacological activity in mammals (EMA, 2009).

Single dose toxicity was studied in rats with a limit dose of 2000 mg/kg applied orally and dermally, and monepantel was found to be of low acute toxicity without mortality or treatment-related effects on respiration rate, body temperature, blood pressure or heart rate.

Repeat dose toxicity was investigated in rats, mice and dogs; in all studies, monepantel was seen to be well tolerated clinically. The rat studies were conducted by dietary administration of monepantel with target high doses of 1000 mg/kg in the 4-week, 900 mg/kg in the 13-week and about 700 mg/kg in the 52-week study, respectively. Main targets of toxicological significance were the lipid metabolism, with increased cholesterol, triglycerides and phospholipids, and the liver with increased organ weight, associated with centrilobular hepatocellular hypertrophy, ob served at the higher dose levels. NOELs of 15 and 14 mg/kg were determined in the 13- and 52-week studies, respectively, corresponding to a human equivalent dose (HED) of about 2.2 – 2.4 mg/kg or about 140 mg. No histopathological lesions of major toxicological concern were detected, and, specifically, there was no indication of any proliferative lesions in the 52-week study. Similar results were obtained in a 13-week study in mice at target doses up to 1000 mg/kg administered in the feed. No treatment-related clinical signs were seen, and only some clinical chemistry parameters, especially liver enzymes, showed changes. In this study, too, histopathological changes were confined to centrilobular fatty change in the liver. A NOAEL of 18 mg/kg was determined resulting in a HED of about 1.5 mg/kg.

In dogs, exposed to monepantel through the feed, similar effects could be observed. In a 4-week study, target doses of up to 1217/1472 mg/kg (for males/females) were administered. The main effects were increased alkaline phosphatase activities and increased adrenal weights at all doses, and lower thymus weight, thymus involution and increased liver weight (in females only) were seen at the high dose. The liver weight increase in females was without a histopathological correlate. In a subsequent 13-week study with doses of up to 963/1176 mg/kg (for males/females) analogous observations were made: Treatment related changes in alkaline phosphatase values were noted in all groups, but alanine transaminase and aspartate transaminase were not affected. In the highest dose group in females, gamma-glutamyl transpeptidase was increased. Higher liver weights were recorded at all concentrations in both sexes at the end of the treatment period. Histopathologically, treatment-related changes were noted in the liver (hepatocellular hypertrophy, biliary proliferation, brown pigments in Kupffer cells and hepatocytes), small intestine (dilatation of glands) and pancreas (increased apoptosis) in the various groups. The bile duct proliferation, the brown pigment in hepatocytes/Kupffer cells and the increase in alkaline phosphatase activity could all be related to potential liver cholestasis. Finally, in the 52-week study, doses of up to 91/99 mg/kg (for males/females) were administered, leading to essentially the same findings as in the shorter-term studies. The effects seen on alkaline phosphatase activity and liver weights associated with hepatocellular hypertrophy indicated the liver as the main target organ. Histopathology showed bile duct hyperplasia in the liver of both sexes at the high dose, while other histopathological findings were observed at all doses including controls. No proliferative pre-neoplastic lesions were observed. After withholding treatment effects were seen to decrease (although not to control levels at 4 weeks) indicating that the changes are reversible. While a dose related response was evident for the liver effects, particularly the increase in alkaline phosphatase activity, the overall conclusion from the statistical analyses (using analysis of variance) was that the effects were not statistically significant at the lowest dose of 3 mg/kg, establishing this dose as the NOAEL, corresponding to an HED of about 1.6 mg/kg or 100 mg for a 60 kg adult.

Reproductive toxicity studies were conducted in rats and rabbits and provided no evidence for foetal toxicities (embryotoxicity, foetotoxicity, teratogenicity) up to maternal doses of 1000 mg/kg, and showed no reproductive toxicity effects in a 2-generation study in rats up to a dose of 200 mg/kg.

In the standard test package of genotoxicity studies, monepantel did not provide any evidence of mutagenic activity.

Monepantel was also tested for carcinogenicity in two conventional life-time studies in rats and mice. In rats, treated with monepantel at target doses of up to 578/707 mg/kg (for males/females) in the feed, no specific clinical signs could be observed and linked with the treatment. Slightly increased liver, kidney and heart weights were recorded in females fed the two upper dose levels, but, in the absence of any correlating microscopic findings, these effects were considered to be probably non-adverse. The incidence, onset and location of palpable nodules/masses did not distinguish test item-treated rats from their respective controls. Similarly, the incidence of histopathological lesions did not indicate any effect of treatment, and monepantel was thus not considered to be carcinogenic in rats. The same conclusion was reached in the study in mice.

Finally, tests for skin sensitization, dermal and eye irritation, and delayed hypersensitivity provided no evidence for any major reasons for concern with respect to these properties (EMA, 2009).

Genotoxicity information has also been reported in a publication. No effects were observed in the Ames test at concentrations of up to 5000 µg/plate, in a chromosomal aberration assay in vitro at concentrations of up to 140 µg/ml, both assays with and without exogenous metabolic activation, as well as in an in vivo mouse bone marrow micronucleus test. Monepantel was thus declared to be without mutagenic or genotoxic potential (Kaminsky et al., 2008b).

Reproductive toxicity was furthermore studied in sheep. Rams were given repeated oral doses of monepantel of 11.25 mg/kg, i.e., three times the maximum recommended dose, every 5 days over an entire spermatogenic cycle and during mating for a total of 100 days. Detailed recording at multiple time points were made of veterinary examinations; observations for adverse events; bodyweight measurements; faecal scores; haematology, clinical chemistry and coagulation variables; semen indices; evaluation of serving capacity; and gross pathology (including measurement of organ weights) performed on 10 rams from each group at the completion of the study. Monepantel was systemically very well tolerated and was not associated with any treatment-related adverse effects on the reproductive performance (Malikides et al., 2009a). Similarly, ewes were treated in the same way with the same dose and for the same length of time. All ewes treated with monepantel and those in the control group thrived and behaved normally to the end of the study. No treatment-related, toxicologically relevant adverse events, clinical observations or gross post-mortem changes were observed. Furthermore, there were no significant differences in bodyweight or organ weights, and haematological, clinical chemistry or coagulation variables between ewes treated with monepantel and control ewes. No significant differences were observed in any of the reproductive indices measured. No significant clinical differences were noted between lambs born from treated ewes and those from controls (Malikides et al., 2009b). Finally, weaned lambs were given doses of 3.75 mg/kg, 11.25 mg/kg and18.75 mg/kg orally on eight occasions at intervals of approximately 21 days. Detailed recording at multiple time points were made of veterinary examinations, observations for adverse events, bodyweight measurements, faecal scores, and haematology, clinical chemistry and coagulation variables. Gross pathology (including measurement of organ weights) and histopathology were performed at the completion of the study. All lambs grew and behaved normally to the end of the study. No treatment-related, toxicologically relevant adverse events, clinical observations or macroscopic or microscopic changes were observed. Furthermore, there were no significant differences in bodyweight or organ weights, and haematological, clinical chemistry or coagulation variables between lambs treated with monepantel and control lambs (Malikides et al., 2009c).

**Conclusions and Recommendations**

Monepantel is a new anthelminthic with a new mode of action different from those of other anthelminthics which makes it active against nematodes which are resistant to current drugs. It inhibits the activity of a nematode-specific subunit of the nicotinic acetylcholine receptor which is not present in mammals, and thus combines high anti-parasitic efficacy with low mammalian toxicity. A single dose of 2.5 mg/kg is very efficacious in sheep, a dose which would not be far away from the human equivalent dose. The main toxic effects observed in non-clinical studies are on the liver, although the findings do not conform to serious hepatotoxicity but are rather to be viewed as adaptive responses.

For the conduct of clinical trials, i.e., for the submission of an IND, the presently available safety information would seem to be sufficient. The activity of the sulfone metabolite should, however, be clarified. Protein binding of monepantel is high, and this might cause problems with drug-drug interactions; however, no data are available for the protein binding properties of the sulfone metabolite, which might alleviate the problem somewhat, provided its binding were lower than the one of the parent substance. This should also be clarified. Safety pharmacology and toxicology do not point to major safety concerns, and even though the HEDs of the various NOAELs are at similar dose levels, they derive from repeat dose studies, while in the veterinary clinical application only a single dose is administered. The non-clinical toxicology data thus do not provide clues as to the possibility of serious adverse events being induced by monepantel, since the main toxicological effects, namely those on the liver, seem to be more of an adaptive than a toxic nature. This adaptive response, however, could point to a cytochrome P450-inducing property of monepantel, and this should certainly be investigated and taken into account when treating patients who may possibly be also under additional medication.

The Lazar Toxicity Prediction software, which compares an unknown agent in a structure-activity relationship to the maximum recommended daily doses (MDD) of pharmaceuticals in the FDA database, provides a value of 0.0255 mM/kg/day, equal to a dose of 12 mg/kg/day. Although the confidence value placed on the calculated MDD is with 5% rather low, it may nevertheless be considered as a valid estimate.

One advantage of a possible use of monepantel in the context of anthelminthic treatments in disease-endemic countries would most probably be its single-dose regimen, provided the efficacy of a single dose can be reproduced and proven in humans.

In conclusion, the development of monepantel for the treatment of human nematode infections should be seriously considered.

**References**

EMA European Medicines Agency (2009). European Public Assessment Report (EPAR) on Zolvix® (Monepantel). Downloadable from: http://www.ema.europa.eu/vetdocs/vets/Epar/zolvix/zolvix.htm

Hosking BC, Stein PA, Mosimann D, Seewald W, Strehlau G, Kaminsky R (2008). Dose determination studies for monepantel, an amino-acetonitrile derivative, against fourth stage gastro-intestinal nematode larvae infecting sheep. Vet Parasitol 157: 72-80.

Hosking BC, Griffiths TM, Woodgate RG, Besier RB, Le Feuvre AS, Nilon P, Trengove C, Vanhoff KJ, Kaye-Smith BG, Seewald W (2009a). [Clinical field study to evaluate the efficacy and safety of the amino-acetonitrile derivative, monepantel, compared with registered anthelmintics against gastrointestinal nematodes of sheep in Australia.](http://www.ncbi.nlm.nih.gov/pubmed/19857240?itool=EntrezSystem2.PEntrez.Pubmed.Pubmed_ResultsPanel.Pubmed_RVDocSum&ordinalpos=2) Aust Vet J 87(11):455-62.

Hosking BC, Dobson DP, Stein PA, Kaminsky R, Bapst B, Mosimann D, Mason PC, Seewald W, Strehlau G, Sager H (2009b). Dose confirmation studies for monepantel, an amino-acetonitrile derivative, against fourth stage gastro-intestinal nematode larvae infecting sheep. Vet Parasitol 160: 251-257.

Hosking BC, Kaminsky R, Sager H, Rolfe PF, Seewald W (2010). A pooled analysis of the efficacy of monepantel, an amino-acetonitrile derivative against gastrointestinal nematodes of sheep. Parasitol Res 106: 529-532.

Kaminsky R, Ducray P, Jung M, Clover R, Rufener L, Bouvier J, Weber SS, Wenger A, Wieland-Berghausen S, Goebel T, Gauvry N, Pautrat F, Skripsky T, Froelich O, Komoin-Oka C, Westlund B, Sluder A, Mäser P (2008a). [A new class of anthelmintics effective against drug-resistant nematodes.](http://www.ncbi.nlm.nih.gov/pubmed/18337814?itool=EntrezSystem2.PEntrez.Pubmed.Pubmed_ResultsPanel.Pubmed_RVDocSum&ordinalpos=12) Nature 452(7184): 176-80

Kaminsky R, Gauvry N, Schorderet WS, Skripsky T, Bouvier J, Wenger A, Schroeder F, Desaules Y, Hotz R, Goebel T, Hosking BC, Pautrat F, Wieland-Berghausen S, Ducray P (2008b). Identification of the amino-acetonitrile derivative monepantel (AAD 1566) as a new anthelminthic drug development candidate. Parasitol Res 103: 931-939.

Kaminsky R, Mosimann D, Sager H, Stein P, Hosking B (2009). Determination of the effective dose rate for monepantel (AAD 1566) against adult gastro-intestinal nematodes in sheep. Int J Parasitol 39: 443-446.

Karadzovska D, Seewald W, Browning A, Smal M, Bouvier J, Giraudel JM (2009). Pharmacokinetics of monepantel and its sulfone metabolite, monepantel sulfone, after intravenous and oral administration in sheep. J Vet Pharmacol Ther 32: 359‑367.

Malikides N, Spencer K, Mahoney R, Baker K, Vanhoff K, Hall C, Debenedetti R, Strehlau GA (2009a). Safety of an amino-acetonitrile derivative (AAD), monepantel, in ewes and their offspring following repeated oral administration. N Z Vet J 57: 193-202.

Malikides N, Helbig R, Roth DR, Alexander A, Hosking BC, et al. (2009) Safety of an amino-acetonitrile derivative ( AAD ), monepantel, in weaned lambs following repeated oral administration. N Z Vet J 57: 10-15.

Malikides N, Helbig R, Mahoney R, George B, Baker K, Vanhoff K, Spencer K, Hall C, Debenedetti R, Strehlau GA (2009b). [Reproductive safety of an amino-acetonitrile derivative ( AAD ), monepantel, in rams following repeated oral administration.](http://www.ncbi.nlm.nih.gov/pubmed/19252538?itool=EntrezSystem2.PEntrez.Pubmed.Pubmed_ResultsPanel.Pubmed_RVDocSum&ordinalpos=2) N Z Vet J. 57(1):16-21.

Mason PC, Hosking BC, Nottingham RM, Cole DJ, Seewald W, McKay CH, Griffiths TM, Kaye-Smith BG, Chamberlain B (2009). A large-scale clinical field study to evaluate the efficacy and safety of an oral formulation of the amino-acetonitrile derivative (AAD ), monepantel, in sheep in New Zealand. N Z Vet J 57: 3-9.

Rufener L, Maser P, Roditi I, Kaminsky R (2009). *Haemonchus contortus* acetylcholine receptors of the DEG-3 subfamily and their role in sensitivity to monepantel. PLoS Pathog 5(4): 1-11.

Sager H, Hosking B, Bapst B, Stein P, Vanhoff K, Kaminsky R (2009). Efficacy of the amino-acetonitrile derivative, monepantel, against experimental and natural adult stage gastro-intestinal nematode infections in sheep. Vet Parasitol 159: 49‑54.
