# Supplementary material for: Potential Drug Development Candidates for Human Soil-Transmitted Helminthiases
Source: PLoS Negl Trop Dis. 2011 Jun 7;5(6):e1138. doi: 10.1371/journal.pntd.0001138 (PMC3111745; doi:10.1371/journal.pntd.0001138)
Supplement: Appendix S6 — Detailed information on nitazoxanide. (DOC) [file pntd.0001138.s006.doc]

**Summary Report on**

**Nitazoxanide (Alinia®)**

**General Information**

Nitazoxanide, with the chemical name 2-(acetyloxy)-N-(5-nitro-2-thiazolyl)benz-amide (MW 307.29; CAS No. 55981-090-4; see chemical structure below) is a new antiprotozoal drug, registered in the United States since 2002 and marketed by Romark Laboratories as a human pharmaceutical under the trade name Alinia®. It is also widely available in South America under a number of different trade names (White, 2004). It is indicated for the treatment of diarrhoea caused by *Giardia lamblia* and of diarrhoea caused by *Cryptosporidium parvum*. The recommended dosage is 100 mg (1 – 3 years), 200 mg (4 – 11 years) and 500 mg (≥ 12 years) every 12 hours for 3 days (FDA label).

Its anthelminthic properties had been noticed already early on, and the compound was marketed in the 70ies and early 80ies by the Institut Mérieux under the trade name Taenitaz for the control of cestodes in cats and dogs (Murphy and Friedman, 1984). At this time already, its activity against a number of additional gastrointestinal helminths was demonstrated in clinical trials, although non-inferiority against the standard treatments with albendazole or praziquantel could not be shown (Anderson and Curran, 2007), which was probably the reason why the compound was ultimately developed only for the antiprotozoal indications listed above.

Besides the information available for nitazoxanide in the various FDA Reviews, there are some current reviews on its properties (White, 2004; Fox and Savarolatz, 2005; Anderson and Curran, 2007; van den Enden, 2009), as well as a number of publications dealing with specific aspects of the compound. These latter reports concentrate mainly on efficacy aspects of the drug, while for the non-clinical safety issues the FDA approval documents are the only source of information.

**Nitazoxanide Mechanism of Action**

The mechanism of action of nitazoxanide and its active metabolite, desacetyl-nitazoxanide or tizoxanide, has been elucidated primarily for its protozoan indications. The survival of protozoa under anaerobic conditions, such as are present in the gastrointestinal tract, depends on the enzyme pyruvate:ferredoxin oxidoreductase which oxidizes pyruvate to acetylCoA using ferredoxin as an electron acceptor *in vivo*. Nitazoxanide and tizoxanide can act as alternate electron acceptors for this enzyme, and it was thus hypothesized that in this process a toxic radical may be generated which could be considered as the ultimate toxic product (FDA, 2004).

More recently, nitazoxanide has been shown to be a non-competitive inhibitor of the pyruvate:ferredoxin/flavodoxin oxidoreductases (PFORs) of a number of bacteria, protozoa and helminths, such as *Trichomonas vaginalis, Entamoeba histolytica, Giardia intestinalis, Clostridium difficile, Clostridium perfringens, H. pylori, and Campylobacter jejuni*. Under the influence of nitazoxanide the pyruvate-dependent PFOR activity did not result in the accumulation of products (acetyl coenzyme A and CO_2_) and pyruvate was not consumed in the reaction. It was thus proposed that nitazoxanide should intercept PFOR at an early step in the formation of the lactyl-TPP transition intermediate, resulting in the reversal of pyruvate binding prior to decarboxylation and in coordination with proton transfer to nitazoxanide. Thus, nitazoxanide might be the first example of an antimicrobial drug that targets the “activated cofactor” of an enzymatic reaction rather than its substrate or catalytic sites, a novel mechanism that may escape mutation-based drug resistance (Hoffman et al., 2007). Nitazoxanide did also act as electron acceptor in the PFOR catalyzed reaction in *Entamoeba histolytica, Trichomonas vaginalis, Clostridium sp.,* and *Helicobacter pylori* (FDA, 2004). As an additional target in the mitochondrial respiratory chain the NADH-fumarate reductase system has been proposed. In *Echinococcus multilocularis*, the parasite responsible for alveolar echinococcosis, this system has been shown to be responsible for the high adaptation of the parasite to anaerobic environments. In vitro assays using respiratory chain inhibitors against this system demonstrated that they had a potent ability to kill *Echinococcus multilocularis* protoscoleces, suggesting that the mitochondrial respiratory chain of this parasite could be a target for nitazoxanide action (Matsumoto et al., 2008).

Also, nitroreductases have been thought to be involved in the toxic action of nitazoxanide. A nitroreductase has been isolated from cell extracts of *Giardia lamblia* trophozoites which was overexpressed in Escherichia coli, purified, and then characterized using an assay for oxygen-insensitive nitroreductases with dinitrotoluene as a substrate. In this assay system, nitroreductase activity was severely inhibited by nitazoxanide, demonstrating that the antigiardial activity of this drug could be, at least partially, mediated also through the inhibition of nitroreductases, again important for the survival of these organisms in an anaerobic environment (Müller et al., 2007a).

Additionally, also protein disulphide isomerases (PDI) have been identified as potential targets of nitazoxanide activity. In *Giardia intestinalis*, which expresses five PDI variants, PDI2 and PDI4 expression was shown to be significantly downregulated during in vitro treatment with nitazoxanide, indicating that the drug is targeting this enzyme (Müller et al., 2007b). The *in vitro* activity of nitazoxanide against *Neospora caninum* (Esposito et al., 2005) was later demonstrated to be related to an inhibition of protein disulphide isomerase (NcPDI). Binding of tizoxanide and nitazoxanide led to the inhibition of its function by these and other thiazolides (Müller et al., 2008a). Additionally, recombinant PDI2 and PDI4 were shown to be inhibited by nitazoxanide in a similar concentration range as rec-NcPDI (Müller et al., 2008b).

**Non-Clinical Efficacy Data**

The major part of the regulatory non-clinical efficacy information is dealing with the two protozoa, *Giardia lamblia* and *Cryptosporidium parvum*, as the basis for the approved indications. These data, as well as published literature references, will not be summarized here, since the therapeutic usefulness of nitazoxanide in the approved indications and dosage can be considered proven. The FDA approval reviews contain, however, also some information on the activities of nitazoxanide against other parasites, and a number of publications are additionally available dealing with the efficacy of nitazoxanide against a variety of parasites.

The activities of nitazoxanide and tizoxanide against *Trichomonas vaginalis* were determined *in vitro* in two tests and compared to the respective activity of metronidazole. In one assay, the minimum lethal concentrations of nitazoxanide and tizoxanide were about 8-fold lower than the metronidazole values, while in the other test, metronidazole was more active by a factor of 4 than nitazoxanide. In an *in vivo* assay with infected rats, nitazoxanide was less active than metronidazole when administered orally, with a dose of 200 mg/kg producing similar effects to a metronidazole dose of 20 mg/kg and being less effective than a metronidazole dose of 50 mg/kg. On the other hand, nitazoxanide, at a dose of 5 mg administered locally into the vagina, showed an activity equal to the same dose of metronidazole (FDA, 2004). Also, nitazoxanide was found to be of similar activity *in vitro* against *Entamoeba histolytica*, when compared to metronidazole.

Mature flukes of *Fasciola hepatica* and *Fasciola gigantea* were exposed to nitazoxanide *in vitro*, and concentrations of ≥10 µg/ml were reported to result in decreased motility and increased mortality compared to controls. Two in vivo studies in rabbits infected with *Fasciola gigantea* metacercariae investigated the efficacy of a nitazoxanide treatment (35 and 75 mg/kg for 7 days) on the number of immature and mature flukes in liver tissue and in the abdominal cavity and intestinal surfaces. When treatment was directed against immature stages (start of treatment 4 weeks after infection) a 40 to 60 % reduction was reported, whereas treatment initiated10 weeks after infection resulted in complete eradication of mature flukes (FDA, 2004).

No *in vitro* studies of nitazoxanide activity against nematodes and cestodes were submitted in the FDA file. A number of *in vivo* studies are included in the FDA file which, however, were mostly difficult to interpret due to the lack of untreated controls and the lack of raw data. Therapeutic activity is reported for single up to three doses of up to 200 mg/kg against *Taenia pisiformis* and *Dipyllidium caninum* in dogs, *Taenia taeniaeformis* in cats, and different tapeworms in sheep. Experimental studies were conducted in mice infected with *Syphacia obvelata* and *Hymenolepsis nana* and treated with nitazoxanide at a dose of 200 mg/kg for 4 days. Partial activity was reported against *Syphacia obvelata*, while complete deparasitization was observed for *Hymenolepsis nana*. In an additional study with *Hymenolepsis nana* in mice, the lowest dose of nitazoxanide effective in clearing worms from the small intestine was determined to be ≥50 mg/kg (FDA, 2004).

From published literature, the following information about efficacy of nitazoxanide against organisms other than *Giardia* and *Cryptosporidium* can be extracted:

The potential nematocidal effect of nitazoxanide against *Caenorhabditis elegans*, *Heligmosomoides polygyrus* and *Trichinella spiralis* was studied *in vitro* and *in vivo* in comparison to mebendazole, albendazole and levamisole. Nitazoxanide concentrations of 50 and 100 µM were needed *in vitro* to demonstrate low activity, while the comparators proved active in concentrations of 1 to 10 µM. Also when tested in infected mice *in vivo*, doses of up to 1000 mg/kg nitazoxanide were inactive (*T. spiralis*) or at most slightly active (*H. polygyrus*), while the comparators showed good activities (Fonseca-Salamanca et al., 2003).

The efficacy of nitazoxanide and its active metabolite tizoxanide against another nematode, *Brugia malayi*, was studied *in vitro* and *in vivo*. *In vitro*, both compounds reduced worm motility and viability in a concentration-dependent manner. Worm viability was reduced by 50% with both compounds at 2.5 µg/ml, and 20 µg/ml killed adult worms; at 5 µg/ml microfilaria release was significantly reduced. These compounds blocked embryogenesis, and decreased microfilarial motility and viability. Neither of the compounds when administered orally, subcutaneously or intraperitoneally at a dose of 100 mg/kg for 5 days, however, cleared adult worms or microfilariae in infected gerbils; in contrast, subcutaneous albendazole treatment at this same dose and regimen completely cleared worms. These results indicate that both, nitazoxanide and tizoxanide, although having potent effects on *B. malayi* when tested *in vitro*, are ineffective *in vivo* (Rao et al., 2008).

The efficacy of nitazoxanide against metacestodes of *Echinococcus multilocularis* was investigated *in vitro*. Treatment at concentrations of 1, 5 and 10 µg/ml induced high levels of alkaline phosphatase activity in the medium. Concurrently, distinct morphological and ultrastructural alterations were detected in the treated metacestodes in comparison to untreated controls. Their nonviability was subsequently confirmed through bioassay, i.e., inoculation of treated and untreated parasites into mice, demonstrating the *in vitro* parasiticidal effect of nitazoxanide on *E. multilocularis* metacestodes (Stettler et al., 2003). Nitazoxanide was tested along with other drugs for their efficacy against *E. multilocularis* larvae that had been grown intraperitoneally in Mongolian gerbils and then transferred into tissue culture. After 6 weeks in culture drugs were added and the effect on the vesicles that had budded from the tissue blocks was observed. At high nitazoxanide concentrations (10 µg/ml), disintegration of all vesicles was observed after 7 days and was significantly more rapid than with albendazole at equal concentrations (21 days). After drug discontinuation, regrowth of vesicles, however, occurred between 7 and 14 days, indicating a parasitostatic effect. Combination treatment with nitazoxanide-albendazole at concentrations between 1 and 10 µg/ml for either 3 weeks, 3 months, or 6 months yielded no vesicle regrowth during 8 months after drug discontinuation. The treated larval tissue was injected intraperitoneally into gerbils, and no regrowth of larval tissue was observed, suggesting a parasitocidal effect after combined treatment (Reuter et al., 2006). Nitazoxanide proved also to be active *in vitro* against protoscoleces as well as metacestodes of *E. granulosus*. Concentration-dependent mortality of protoscoleces was observed at concentrations of 1 to 10 µg/ml, with death of these stages confirmed by subsequent further *in vitro* culture. Metacestodes were killed by a nitazoxanide concentration of 10 µg/ml, an effect comparable to the activity of albendazole (Walker et al., 2004).

The *in vitro* efficacy of nitazoxanide, its active metabolite tizoxanide as well as a combination of tizoxanide and albendazole sulphoxide was tested against *Taenia crassiceps* cysts. Nitazoxanide and tizoxanide exhibited cestocidal activity which was time- and concentration-dependent with EC_50_ values of 0.15, 0.12 and 0.080 µg/mL for nitazoxanide, tizoxanide and albendazole sulphoxide, respectively. No statistical differences between EC_50_ values were found, indicating that nitazoxanide and tizoxanide are equally potent as albendazole sulphoxide. The effect of the tizoxanide and albendazole sulphoxide combination was faster than that observed with each drug alone. In the opinion of the authors, these results suggested that nitazoxanide in combination with albendazole could be useful for treatment of cysticercosis infections, although confirmation of the activity would be needed in an *in vivo* model (Palomares-Alonso et al., 2007).

The susceptibility of *Toxocara canis* to nitazoxanide was investigated in a mouse model in comparison to albendazole and ivermectin. In this model, mice were each infected with 500 embryonated eggs of *T. canis* and treated, beginning on the second day after infection, with either a single 8 mg dose of albendazole every 12 h for a 10-day period, three 2 mg doses of ivermectin given every 5 days, or a single 2.5 mg dose of nitazoxanide every 12 hours for 3 days. In all these procedures, administration of the corresponding drug reduced the number of larvae that reached the brain and other encephalic areas. The reduction in the mean number of live larvae found was significantly lower for the nitazoxanide treatment in comparison to controls, with similar susceptibilities observed in the albendazole and ivermectin treatments. Not only the number of live larvae but also their motility was significantly reduced in all treatments. Thus, the results of this study showed that nitazoxanide, similar to albendazole and ivermectin, is able to reduce the number of *T. canis* larvae that reach the central nervous system in the experimental toxocariasis rodent model (Delgado et al., 2008).

One of the major problems associated with the lack of newer anthelminthic drugs is the possibility of emerging resistance towards the existing, older drugs. In this respect, it should be important that such resistant parasites would not be cross-resistant to the newer drugs.

Two strains of *Giardia lamblia* were generated that exhibited resistance to either nitazoxanide or metronidazole and their susceptibilities to both drugs were determined. Using quantitative RT–PCR, the expression of genes that are potentially involved in resistance formation, namely genes encoding pyruvate oxidoreductases (POR1 and POR2), nitroreductase (NR), protein disulphide isomerases (PDI2 and PDI4) and variant surface proteins (VSPs; TSA417) were analysed. Whereas one of the two strains was cross-resistant to nitazoxanide and to metronidazole, the other was resistant only to metronidazole. Transcript levels of the potential targets for nitro-drugs, i.e., POR1, POR2 and NR, were only slightly modified; however, PDI2 transcript levels were increased in both resistant strains and PDI4 levels in the metronidazole-resistant strain. This correlated with the findings that the functional activities of recombinant PDI2 and PDI4 were inhibited by nitazoxanide. These results thus suggest that resistance formation in *Giardia* against nitazoxanide and metronidazole is linked (Müller et al., 2007a).

**Non-Clinical ADME Data**

There are no published data on pharmacokinetic and ADME properties of nitazoxanide with the exception of an *in vitro* study on intestinal epithelial transfer of nitazoxanide (Matysiak-Budnik et al., 2002). In this latter publication, an important transepithelial transport of nitazoxanide across a monolayer of HT29‑19A intestinal epithelial cells with a rapid, although transitory intracellular accumulation of the drug was observed, with no difference between the apical to basal and basal to apical fluxes of the drug.

Animal kinetic and ADME studies summarized in the FDA Review indicate that nitazoxanide is rapidly converted in biological fluids to its desacetylated metabolite tizoxanide, most probably by the combined action of nonspecific esterases and spontaneous (chemical) hydrolysis. No extensive metabolic studies were conducted, however, and only glucuronidated tizoxanide was confirmed as a further metabolite of nitazoxanide. In the dog, radioactivity from a nitazoxanide dose is excreted to about 26% in the urine and 46% in the faeces. *In vitro* studies indicated no potential of tizoxanide to interact with cytochrome P450 enzymes, where IC_50_ values were determined which are in clear excess of the maximum plasma concentrations achieved in the human therapeutic situation. Specifically, CYP2C9 and CYP3A4 which are highly involved in drug metabolism were inhibited by tizoxanide at IC_50_ concentrations of >100 µM and 2600 µM, respectively, as compared to a C_max_ of about 40 µM after a single oral dose of 500 mg in humans. The high protein binding of >99% has, however, to be taken into account as a potential source of drug-drug interactions, especially for such drugs that are also highly protein-bound and which exhibit narrow safety windows (FDA, 2004).

**Non-Clinical Safety Data**

No information on safety pharmacology and toxicology of nitazoxanide is available in the published literature, except an early report on some toxicological properties of nitazoxanide (Murphy and Friedman, 1985). Therefore, the safety assessment of nitazoxanide has to be relied practically exclusively on the summaries presented in the FDA review files. These summaries are provided in the Attachment.

In the paper by Murphy and Friedman (1984) acute and chronic toxicity were studied in mice, rats, dogs and cats, genetic toxicity was investigated by means of the Ames test, and also primary eye irritation (Draize test) was looked for. In all species acute toxicity was very low, mice being the most sensitive species with an oral LD_50_ value of about 1350 – 1380 mg/kg (males and females, respectively), while rats, dogs and cats tolerated oral doses of 10 g/kg with only minor signs of toxicity and no mortality. There was no mutagenic activity observed in the Ames test, and the compound was judged non-irritating for the eye. In the 14-week repeat dose toxicity study in rats oral doses of 50, 150 and 450 mg/kg were applied, with the lower doses showing no treatment-related effects. Other findings were restricted to slightly lower red cell parameters (erythrocyte counts and haematocrit) at the high dose in females and to stomach ulcerations in males and females of the two higher dose groups.

The summaries provided in the FDA Review corroborate and extend these findings and conclusions (FDA, 2004). Briefly, the more extensive studies summarized therein on safety pharmacology, general toxicology, genetic toxicology and reproductive toxicology did not provide evidence for any major safety problems associated with the human use of nitazoxanide at recommended doses. The only findings with potential consequences in terms of possible side effects are 1) the irritative potential of the drug to the gastrointestinal tract which is possibly mediated by its conversion to salicylate-like metabolites, and 2) its propensity to induce anaemia possibly related to the presence of a nitro group in the molecule.

**Conclusions and Recommendations**

The successful application of oral nitazoxanide for the treatment of diarrhoeas caused by *Giardia lamblia* and *Cryptosporidium parvum* and its broad spectrum of activity seen in a number of *in vitro* investigations have prompted the search for additional indications in the area of protozoan and helminthic infections. Nitazoxanide appears to be a drug with no major safety issues emerging from non-clinical safety pharmacology and toxicology studies. The haematotoxicity observed in rats and dogs with extramedullary haematopoiesis and reduced erythrocyte counts and haematocrit may be considered as related to the presence of a nitro-group in the molecule. Consequently, the use of nitazoxanide in G6PD-deficient patients might warrant special consideration. The main toxic finding with immediate relevance for the clinical situation is the irritative effect on the gastrointestinal epithelia which may eventually lead to gastrointestinal bleeding and ulceration. This effect, however, has been observed in long-term repeat dose toxicity studies, and the recommended 3-day course of treatment might not be of sufficient length to induce the respective side-effects in patients.

In terms of the activity spectrum, it appears clear from non-clinical *in vitro* and *in vivo* investigations that nitazoxanide is active against a number of protozoa, as well as against a variety of cestodes. These findings have also been corroborated in clinical studies with some of these organisms (Chero et al., 2007; Lateef et al., 2008). Studies conducted with nematodes have resulted in the demonstration of activity *in vitro* which, however, was not reproduced in *in vivo* models. This is in a certain contradiction to clinical experience, where activity against some important human intestinal nematodes has been reported (Romero et al., 1997; Davila-Gutierrez et al., 2002; Diaz et al., 2003; Galvan-Ramirez et al., 2007).

In conclusion, nitazoxanide therefore appears to be a valid candidate for the treatment of a diversity of helminthic diseases. Primarily, the approved dosage regimen should be considered for use in clinical trials. Since the efficacy of nitazoxanide towards certain helminthic infections seemed to be lower than in standard treatments, it could be considered whether increased dosages or increased treatment times, or both should be investigated. The lowest non-clinical NOAEL, extrapolated to a “human equivalent dose” of about 12 mg/kg, lies in the range of the human therapeutic dose of about 11 mg/kg, and higher doses would therefore seem not to be covered by non-clinical safety data. In view of the relatively low incidences of adverse side effects in clinical trials and therapeutic experience which are furthermore mainly mild and transient, however, the human safety of nitazoxanide would not seem to be jeopardized by the use of higher doses and/or extended dosage regimens. Indeed, nitazoxanide dosages up to 1500 mg twice daily have obviously been administered for the recommended duration of three days already, and even for longer periods in patients with protozoal infections and HIV, i.e., for up to 14 days in controlled trials and for up to 1528 days in compassionate use (Anderson and Curran, 2007). It should be possible, therefore, if due consideration is given to the increased probability for the occurrence of gastrointestinal and haematological side effects, to justify such higher doses for the same regimen or even for extended durations of treatment. In these cases, however, patients should be placed under strict surveillance for the above toxicities in small-scale phase II clinical trials.

**References**

Anderson VR, Curran MP (2007). [Nitazoxanide: a review of its use in the treatment of gastrointestinal infections.](http://www.ncbi.nlm.nih.gov/pubmed/17722965?itool=EntrezSystem2.PEntrez.Pubmed.Pubmed_ResultsPanel.Pubmed_RVDocSum&ordinalpos=5) Drugs 67(13):1947-67.

Chero JC, Saito M, Bustos JA, Blanco EM, Gonzalvez G, et al. (2007). *Hymenolepis nana* infection: symptoms and response to nitazoxanide in field conditions. Trans R Soc Trop Med Hyg 101: 203-205.

Davila-Gutierrez CE, Vasquez C, Trujillo-Hernandez B, Huerta M (2002). Nitazoxanide compared with quinfamide and mebendazole in the treatment of helminthic infections and intestinal protozoa in children. Am J Trop Med Hyg 66: 251-254.

Delgado OM, Fernandez G, Silva S, Ramirez O, Romero J, et al. (2008) Preliminary evidence of nitazoxanide activity on *Toxocara canis* in a mouse model. Int J Antimicrob Agents 31: 182-184.

Diaz E, Mondragon J, Ramirez E, Bernal R (2003). Epidemiology and control of intestinal parasites with nitazoxanide in children in Mexico. Am J Trop Med Hyg 68: 384-385.

Esposito M, Stettler R, Moores SL, Pidathala C, Muller N, et al. (2005) In vitro efficacies of nitazoxanide and other thiazolides against Neospora caninum tachyzoites reveal antiparasitic activity independent of the nitro group. Antimicrob Agents Chemother 49: 3715-3723.

FDA (2004). Microbiology, Pharmacology and Clinical Reviews of Alinia, Application number 21-497. Downloadable from: http://www.accessdata.fda.gov/drugsatfda_docs/nda/2004/21-497_21-498s001_Alinia.cfm

Fonseca-Salamanca F, Martinez-Grueiro MM, Martinez-Fernandez AR (2003). Nematocidal activity of nitazoxanide in laboratory models. Parasitol Res 91: 321-324.

[Fox LM](http://www.ncbi.nlm.nih.gov/pubmed?term="Fox LM"%5BAuthor%5D&itool=EntrezSystem2.PEntrez.Pubmed.Pubmed_ResultsPanel.Pubmed_RVAbstract), [Saravolatz LD](http://www.ncbi.nlm.nih.gov/pubmed?term="Saravolatz LD"%5BAuthor%5D&itool=EntrezSystem2.PEntrez.Pubmed.Pubmed_ResultsPanel.Pubmed_RVAbstract) (2005). Nitazoxanide: a new thiazolide antiparasitic agent. [Clin Infect Dis](javascript:AL_get(this, 'jour', 'Clin Infect Dis.');) 40(8):1173-1180.

Galvan-Ramirez ML, Rivera N, Loeza ME, Avila X, Acero J, Troyo R, Bernal R (2007). Nitazoxanide in the treatment of Ascaris lumbricoides in a rural zone of Colima, Mexico. J Helminthology 81: 255-259.

Hoffman PS, Sisson G, Croxen MA, Welch K, Harman WD, Cremades N, Morash MG (2007). Antiparasitic Drug Nitazoxanide Inhibits the Pyruvate Oxidoreductases of Helicobacter pylori, Selected Anaerobic Bacteria and Parasites, and Campylobacter jejuni. Antimicrobial Agents Chemother 51(3): 868–876.

Lateef M, Zargar SA, Khan AR, Nazir M, Shoukat A (2008). Successful treatment of niclosamide- and praziquantel-resistant beef tapeworm infection with nitazoxanide. Int J Infect Dis 12: 80-82.

Matsumoto J, Sakamoto K, Shinjyo N, Kido Y, Yamamoto N, Yagi K, Miyoshi H, Nonaka N, Katakura K, Kita K, Oku Y (2008). Anaerobic NADH-Fumarate Reductase System Is Predominant in the Respiratory Chain of Echinococcus multilocularis, Providing a Novel Target for the Chemotherapy of Alveolar Echinococcosis. Antimicrobial Agents Chemother 52(1): 164–170.

Matysiak-Budnik T, Mégraud F, Heyman M (2002). ln-vitro transfer of nitazoxanide across the intestinal epithelial barrier. J Pharmacy Pharmacol 54: 1413-1417.

Müller J, Wastling J, Sanderson S, Müller N, Hemphill A (2007a). A Novel Giardia lamblia Nitroreductase, GlNR1, Interacts with Nitazoxanide and Other Thiazolides. Antimicrobial Agents Chemother 51(6): 1979–1986.

Müller J, Sterk M, Hemphill A, Müller N (2007b). [Characterization of Giardia lamblia WB C6 clones resistant to nitazoxanide and to metronidazole.](http://www.ncbi.nlm.nih.gov/pubmed/17561498?itool=EntrezSystem2.PEntrez.Pubmed.Pubmed_ResultsPanel.Pubmed_RVDocSum&ordinalpos=4) J Antimicrob Chemother 60(2):280-287.

Müller J, Naguleswaran A, Müller N, Hemphill A (2008a). [Neospora caninum: functional inhibition of protein disulfide isomerase by the broad-spectrum anti-parasitic drug nitazoxanide and other thiazolides.](http://www.ncbi.nlm.nih.gov/pubmed/17720161?itool=EntrezSystem2.PEntrez.Pubmed.Pubmed_ResultsPanel.Pubmed_RVDocSum&ordinalpos=1) Exp Parasitol 118(1):80-88.

Müller J, Ley S, Felger I, Hemphill A, Müller N (2008b). [Identification of differentially expressed genes in a Giardia lamblia WB C6 clone resistant to nitazoxanide and metronidazole.](http://www.ncbi.nlm.nih.gov/pubmed/18408240?itool=EntrezSystem2.PEntrez.Pubmed.Pubmed_ResultsPanel.Pubmed_RVDocSum&ordinalpos=2) J Antimicrob Chemother 62(1):72-82.

Murphy JR, Friedman J-C (1984). Pre-clinical Toxicology of Nitazoxanide – A New Antiparasitic Compound. J Appl Toxicol 5(2):49-52.

Palomares-Alonso F, Piliado JC, Palencia G, Ortiz-Plata A, Jung-Cook H (2007). Efficacy of nitazoxanide, tizoxanide and tizoxanide/albendazole sulphoxide combination against Taenia crassiceps cysts. Journal of Antimicrobial Chemotherapy 59:212–218.

Rao RU, Huang Y, Fischer K, Fischer PU, Weil GJ (2009) Brugia malayi: Effects of nitazoxanide and tizoxanide on adult worms and microfilariae of filarial nematodes. Exp Parasitol 121: 38-45.

Reuter S, Manfras B, Merkle M, Harter G, Kern P (2006) In vitro activities of itraconazole, methiazole, and nitazoxanide versus Echinococcus multilocularis larvae. Antimicrob Agents Chemother 50: 2966-2970.

Romero CR, Guerrero LR, Munoz Garcia MR, Geyne CA (1997). Nitazoxanide for the treatment of intestinal protozoan and helminthic infections in Mexico. Trans R Soc Trop Med Hyg 91: 701-703.

Stettler M, Fink R, Walker M, Gottstein B, Geary TG, Rossignol JF, Hemphill A (2003). In Vitro Parasiticidal Effect of Nitazoxanide against Echinococcus multilocularis Metacestodes. Antimicrob Agents Chemother 47: 467-474.

van den Enden E (2009). [Pharmacotherapy of helminth infection.](http://www.ncbi.nlm.nih.gov/pubmed/19191680?itool=EntrezSystem2.PEntrez.Pubmed.Pubmed_ResultsPanel.Pubmed_RVDocSum&ordinalpos=1) Expert Opin Pharmacother 10(3):435-451.

Walker M, Rossignol JF, Torgerson P, Hemphill A (2004). In vitro effects of nitazoxanide on Echinococcus granulosus protoscoleces and metacestodes. Journal of Antimicrobial Chemotherapy 54:609–616.

White CA Jr. (2004). [Nitazoxanide: a new broad spectrum antiparasitic agent.](http://www.ncbi.nlm.nih.gov/pubmed/15482170?itool=EntrezSystem2.PEntrez.Pubmed.Pubmed_ResultsPanel.Pubmed_RVDocSum&ordinalpos=4) Expert Rev Anti Infect Ther 2(1):43-49.


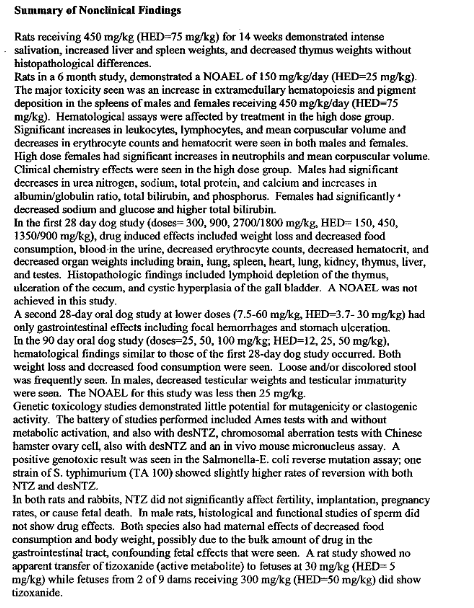

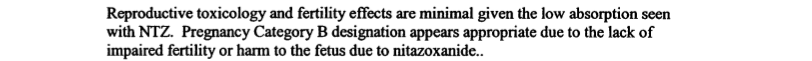

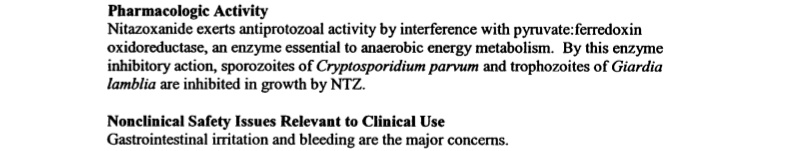
**Attachment: Non-Clinical Safety Summaries from the FDA Review of Alinia**

**
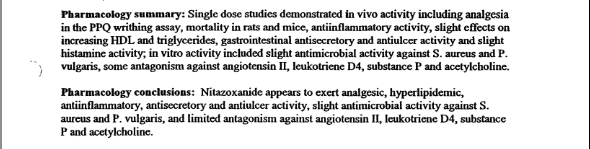

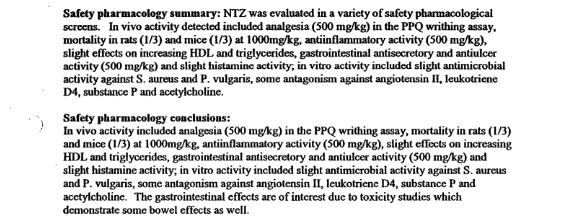

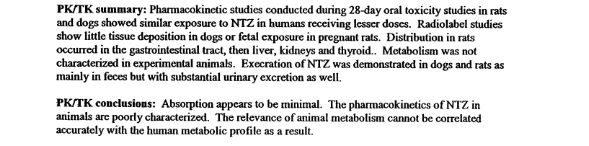

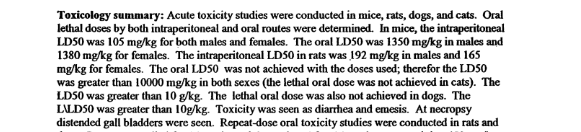
**

**
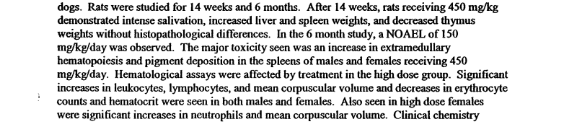

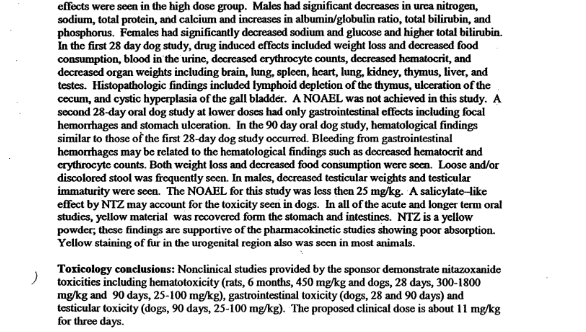

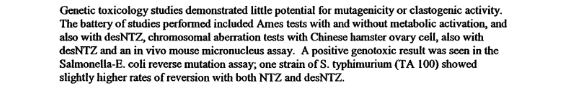
**

**
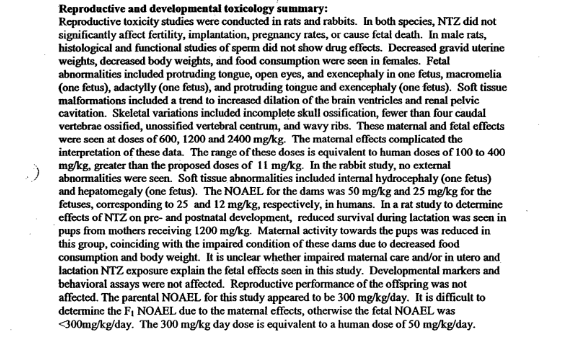
**

**
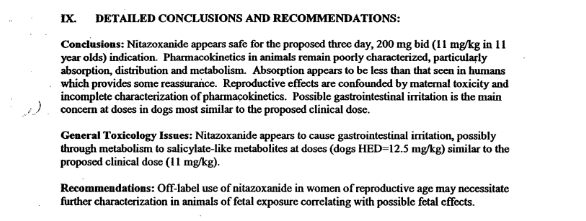
**
